# A novel anti‐c‐Kit antibody–drug conjugate to treat wild‐type and activating‐mutant c‐Kit‐positive tumors

**DOI:** 10.1002/1878-0261.13084

**Published:** 2021-08-29

**Authors:** Jin‐Ock Kim, Kwang‐Hyeok Kim, Eun Ji Baek, Bomi Park, Min Kyung So, Byoung Joon Ko, Han‐Jik Ko, Sang Gyu Park

**Affiliations:** ^1^ College of Pharmacy Ajou University Suwon‐si Korea; ^2^ New Drug Development Center Osong Medical Innovation Foundation Korea; ^3^ School of Biopharmaceutical and Medicinal Sciences Sungshin Women's University Seoul Korea; ^4^ Novelty Nobility Seongnam‐si Korea

**Keywords:** antibody‐drug conjugate, c‐Kit, imatinib‐resistant cancer, stem cell factor, tyrosine kinase inhibitor

## Abstract

c‐Kit overexpression and activating mutations, which are reported in various cancers, including gastrointestinal stromal tumor (GIST), small‐cell lung cancer (SCLC), acute myeloid leukemia, acral melanoma, and systemic mastocytosis (SM), confer resistance to tyrosine kinase inhibitors (TKIs). To overcome TKI resistance, an anti‐c‐Kit antibody–drug conjugate was developed in this study to treat wild‐type and mutant c‐Kit‐positive cancers. NN2101, a fully human IgG1, was conjugated to DM1, a microtubule inhibitor, through *N*‐succinimidyl‐4‐(*N*‐maleimidomethyl) cyclohexane‐1‐carboxylate (SMCC) (to give NN2101‐DM1). The antitumor activity of NN2101‐DM1 was evaluated *in vitro* and *in vivo* using various cancer cell lines. NN2101‐DM1 exhibited potent growth‐inhibitory activities against c‐Kit‐positive cancer cell lines. In a mouse xenograft model, NN2101‐DM1 exhibited potent growth‐inhibitory activities against imatinib‐resistant GIST and SM cells. In addition, NN2101‐DM1 exhibited a significantly higher anti‐cancer effect than carboplatin/etoposide against SCLC cells where c‐Kit does not mediate cancer pathogenesis. Furthermore, the combination of NN2101‐DM1 with imatinib in imatinib‐sensitive GIST cells induced complete remission compared with treatment with NN2101‐DM1 or imatinib alone in mouse xenograft models. These results suggest that NN2101‐DM1 is a potential therapeutic agent for wild‐type and mutant c‐Kit‐positive cancers.

## Graphical abstract

NN2101‐DM1 specifically binds to c‐Kit on surface of cancer cells, including gastrointestinal stromal tumor, small‐cell lung cancer, and systemic mastocytosis. The complex is internalized and degraded by proteases in lysosome, releasing DM1 into cytoplasm. Then, DM1 arrests cell cycle by inhibiting polymerization of microtubule, which results in apoptosis of cancer cell.

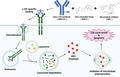

**Abbreviations**
ADC: antibody‐drug conjugate
ADCC: antibody‐dependent cellular cytotoxicity
CDC: complement‐dependent cytotoxicity
DAR: drug‐antibody ratio
GIST: gastrointestinal stromal tumor
HUVEC: human umbilical vein endothelial cell
LAMP‐1: lysosomal‐associated membrane protein 1
SCF: stem cell factor
SCID: severe combined immunodeficiency
SCLC: small‐cell lung cancer
SM: systemic mastocytosis
SMCC: *N*‐succinimidyl‐4(*N*‐maleimidomethyl) cyclohexane‐1‐carboxylate
TGI: tumor growth inhibition
TKI: tyrosine kinase inhibitor

## 1 Introduction

The binding of stem cell factor (SCF) to c‐Kit (also known as CD117) leads to the activation of multiple signaling pathways, including the phosphoinositide 3 kinase, phospholipase C‐gamma, Src kinase, Janus kinase‐signal transducer and activator of transcription, and mitogen‐activated protein kinase pathways [1, 2]. Previous studies have reported that the activated c‐Kit signaling pathway mediates cell growth, migration, and proliferation depending on the cell type. The optimal regulation of the c‐Kit signaling pathway is critical for hematopoiesis, pigmentation, fertility, gut movement, and nervous system functions [3, 4]

c‐Kit kinase activity is dysregulated in various pathological conditions, including cancer, allergy, and vascular leakage [4, 5, 6, 7, 8]. Previous studies have reported that the expression of c‐Kit with oncogenic mutations is dysregulated or upregulated in various cancers, which results in SCF‐independent c‐Kit activation and cell proliferation. Overexpression of c‐Kit is induced by hypoxia as well as activation of various transcription factors, including AP‐2, ETS, SP1, MYB, and MITF [7, 9, 10, 11, 12, 13, 14, 15]. Currently, more than 500 c‐Kit mutations have been identified in human tumors (Sanger Institute Catalogue of Somatic Mutations in Cancer, https://cancer.sanger.ac.uk). However, most of these genetic alterations are silent mutations and not driver mutations. Most oncogenic mutations are located in the juxtamembrane region or within the kinase domain [4]. SCF‐independent spontaneous activating c‐Kit mutations are detected in approximately 85%, 30%, 25%, 25%, and 90% of gastrointestinal stromal tumors (GISTs), acute myeloid leukemias, acral melanomas, testicular carcinomas, and systemic mastocytosis (SM) cases, respectively [4]. However, small‐cell lung cancer (SCLC), which is correlated with smoking, does not harbor oncogenic c‐Kit mutations. Instead, the expression of c‐Kit is reported to be upregulated in 70% of patients with SCLC [16, 17]. Imatinib inhibits the c‐Kit‐induced expression of HIF1A and VEGF in SCLC [18]. However, clinical trials have reported that treatment with imatinib does not result in any clinical benefits in patients with SCLC [18, 19, 20, 21], which suggests that the SCF/c‐Kit pathway is not the major pathway mediating the pathogenesis of SCLC.

Surgical and chemotherapeutic interventions are effective treatments for primary tumors, including breast, ovarian, and lung cancers. However, these interventions do not completely prevent the recurrence of the primary tumor at the same site, which may be because of the presence of therapy‐resistant cancer stem cells. The expression of c‐Kit, a biomarker of cancer stem cells, in ovarian cancer, non‐small cell lung carcinoma, prostate cancer, osteosarcoma, neuroblastoma, and endometrial cancer is correlated with poor prognosis [22, 23, 24, 25, 26, 27, 28, 29, 30, 31, 32]. The SCF/c‐Kit signaling induces expression of ATP‐binding cassette G2 protein related to multi‐drug resistance through β‐catenin and consequently confers resistance to cisplatin, paclitaxel, and imatinib [26]. Additionally, c‐Kit expression is significantly correlated with the development of resistance to platinum‐paclitaxel chemotherapy in patients with cancer. Previous studies have reported that 60% and 10% of c‐Kit‐negative and c‐Kit‐positive malignancies are sensitive to platinum‐paclitaxel chemotherapy, respectively [33]. In particular, the efficacy of small molecules, such as imatinib, to clear cancer stem cells harboring constitutively activating mutations of c‐Kit is limited in tumor cells harboring mutations at different sites. Various small molecules, including dasatinib, sunitinib, and axitinib, have been developed to overcome c‐Kit mutation‐induced therapy resistance [34]. However, the rates of grade 3/4 adverse events in patients treated with these small molecules are higher than those in patients treated with imatinib, limiting their effective application in cancer treatment [35].

Antibody‐drug conjugate (ADC) enables the delivery of potent cytotoxic agents using the specificity of monoclonal antibodies to cancer cells. Recently, various ADCs targeting CD30 (brentuximab vedotin), CD22 (inotuzumab ozogamicin), CD79b (polatuzumab vedotin), and HER2 (trastuzumab emtansine and trastuzumab deruxtecan) have been approved by the US Food and Drug Administration [36]. The development of tyrosine kinase inhibitors (TKIs) is a continuous race against a cyclic process due to treatment‐based continuous gain of mutations in the target molecule. Since ADC is an antibody‐based drug that binds to the extracellular domain of a target molecule, it has the relative advantage of being free from mutations mostly occurring in the intracellular domain, as well as being applicable irrespective of various mutations. Recently, Abrams *et al*. [37] developed ADCs (LOP628) using LMJ729, a humanized anti‐c‐Kit antibody. LMJ729 binds to c‐Kit of humans and monkeys but does not cross‐react with murine c‐Kit. The binding affinity of LMJ729 to c‐Kit was shown to be nanomolar level. Additionally, LMJ729 did not inhibit SCF‐mediated c‐\x8FKit activation, which suggests that target‐mediated adverse events, including neutropenia and reduced hematopoiesis, are minimized as SCF can activate c‐Kit even in the presence of LMJ729. The safety of LMJ729 has been demonstrated in GLP monkey study. However, LMJ729 was associated with hypersensitivity reaction through the enhanced engagement of Fc with Fc receptor in a phase I clinical trial [38]. Previously, we had developed NN2101, which is a fully human antibody targeting c‐Kit that inhibits SCF‐mediated c‐Kit activation by competitively binding with SCF. To reduce the binding affinity of Fc to Fc receptor, a highly fucosylated form of the antibody lacking galactose was designed. NN2101 did not exhibit effector functions [complement‐dependent cytotoxicity (CDC) and antibody‐dependent cellular cytotoxicity (ADCC)]. In this study, NN2101‐DM1, a novel c‐Kit‐targeting ADC, was developed. To generate NN2101‐DM1, DM1 was covalently linked to lysine residues of the NN2101 antibody through *N*‐succinimidyl‐4(*N*‐maleimidomethyl) cyclohexane‐1‐carboxylate (SMCC), which is the same linker payload used in Kadcyla (trastuzumab emtansine), a HER2‐targeting ADC [39, 40]. NN2101 was reported to inhibit SCF/c‐Kit signaling with picomolar binding affinity to c‐Kit [41]. However, the antibody alone cannot inhibit SCF‐independent spontaneous activation induced by c‐Kit mutations, which are reported in various cancers [4]. Hence, in this study, the drug‐to‐antibody ratio (DAR) of NN2101‐DM1 was optimized to be below 2 to minimize c‐Kit‐mediated on‐target adverse effects, including neutropenia and hematopoietic stem cell depletion [37, 38, 42]. NN2101 formed a complex with c‐Kit, and the complex was efficiently internalized and transported into the lysosomes. Additionally, NN2101‐DM1 arrested the cell cycle at G2/M stage and promoted apoptosis of cancer cells. Furthermore, NN2101‐DM1 exhibited potent antitumor activities in mouse xenograft models implanted with wild‐type or mutant c‐Kit‐positive cancer cells. These findings suggest that NN2101‐DM1 is a potential therapeutic agent for various cancers, including GIST, SM, and SCLC.

## 2 Materials and methods

### 2.1 Cell culture

The following cell lines were purchased from the American Type Culture Collection (Manassas, VA, USA): TF‐1, Kasumi‐1, NCI‐H526, NCI‐H889, NCI‐H1048, NCI‐H2170, NCI‐H446, COS7, MDA‐MB‐468, MDA‐MB‐453, MS‐1, SK‐OV‐3, CAOV‐3, and OVCAR‐3. The HMC‐1.2 and human umbilical vein endothelial cells (HUVECs) were purchased from Millipore (Burlington, MA, USA) and Lonza (Basel, Switzerland), respectively. The GIST‐T1, GIST‐430, GIST‐430/654, and GIST‐48 cells were kind gifts from S. Bauer (University Duisburg‐Essen, Germany) and J. A. Fletcher (Brigham and Women's Hospital, Boston, USA). All cells were maintained in a humidified 5% CO_2_ chamber at 37 °C in the respective culture medium, following the manufacturer's instructions.

### 2.2 Antibody preparation

NN2101, a fully human monoclonal IgG1, was prepared as described previously [43]. Briefly, DNA sequences encoding the light and heavy chains were subcloned into the pCHO 1.0 vector (Thermo Fisher Scientific, Waltham, MA, USA). The recombinant vector was transiently transfected into Chinese hamster ovary cells using the ExpiCHO™ expression system kit (Thermo Fisher Scientific), and the cells were cultured for 14 days. NN2101 was purified using AKTA Avant 150 chromatography system (Merck Millipore, Burlington, MA, USA) equipped with MabSelect SuRe™ (Merck Millipore) and HiTrap^®^ Capto™ SP ImpRes (Merck Millipore). The samples were dialyzed against storage buffer (10 mm sodium phosphate, 40 mm NaCl, 0.03% polysorbate 20, and 5% sucrose; pH 6.2). We have previously reported the c‐Kit binding affinity, epitope map, c‐Kit‐positive cell‐binding ability, effector function, immunogenicity, and crystal structure of NN2101 [41].

### 2.3 Enzyme‐linked immunosorbent assay

Recombinant c‐Kit protein (20 ng/100 µL/well; Sino Biological, Beijing, China) was coated onto 96‐well plates (Thermo Fisher Scientific, Roskilde, Denmark) using 1× PBS (pH 7.2) for 12 h at 4 °C. The protein was blocked with 1× PBS containing 5% bovine serum albumin (BSA) at room temperature (RT) for 2 h. NN2101 or NN2101‐DM1 diluted in PBS containing 0.1% Tween‐20 (PBST) and 1% BSA was added to each well and the samples were incubated at RT for 1.5 h. The plate was washed five times with PBST. Next, horseradish peroxidase‐conjugated goat anti‐human IgG (Thermo Fisher Scientific) (1 : 2000) in PBST containing 1% BSA was then added to each well and the plates were incubated at RT for 1.5 h. The plate was washed five times with PBST, and the sample was incubated with 3, 3′, 5, 5′‐tetramethylbenzidine solution (100 µL) for 10 min. The reaction was terminated with 1 m H_2_SO_4_ (50 µL). The absorbance at 450 nm was analyzed using a SPECTROstar Nano Microplate Reader (BMG LABTECH, Ortenberg, Germany).

### 2.4 Western blotting analysis

To analyze the phosphorylation status of signaling mediators induced by SCF or NN2101, the cancer cell lines were starved to minimize the serum‐mediated basal phosphorylation of signaling mediators. Western blotting analysis was performed as described previously [41]. The cells were lysed using radioimmunoprecipitation assay lysis buffer (20 mm Tris‐HCl, pH 7.6, 150 mm NaCl, 1 mm Na_2_EDTA, 1 mm EGTA, 1% NP‐40, 1% sodium deoxycholate, 0.1% sodium dodecyl sulfate (SDS), 10 mm β‐glycerophosphate, 1 mm Na_3_OV_4_, 10 mm NaF, 1 μg·mL^−1^ leupeptin, 1 mm phenylmethylsulfonyl fluoride, 5 μg·mL^−1^ aprotinin, and 2 mm 2‐mercaptoethanol). The proteins were subjected to SDS‐polyacrylamide gel electrophoresis. The resolved proteins were transferred to a polyvinylidene fluoride membrane (Millipore). The membrane was blocked with Tris‐buffered saline (TBS; 20 mm Tris‐HCl, pH 7.4, 150 mm NaCl) containing 5% skim milk and 0.2% Tween‐20 at RT for 1 h. Immunoreactive signals were detected using enhanced chemiluminescence (ECL) solution (Santa Cruz Biotechnology, Dallas, TX, USA). The following antibodies were used in this study: anti‐p‐c‐Kit (Y568/570, Y719, Y703, and Y823; Cell Signaling Technology, Danvers, MA, USA), anti‐c‐Kit (R&D Systems, Minneapolis, MN, USA), anti‐p‐Akt (S473; Cell Signaling Technology), anti‐Akt (Santa Cruz Biotechnology), anti‐p‐ERK1/2 (Cell Signaling Technology), anti‐ERK (Santa Cruz Biotechnology), and anti‐α‐tubulin (laboratory‐made). The short‐interfering RNA (siRNA)‐mediated knockdown of c‐Kit was performed as described previously [44]. Briefly, the cells were transfected with control or c‐Kit siRNA (50 or 200 pmol) using lipofectamine RNAiMAX reagent (Invitrogen, Waltham, MA, USA) for 72 h. The knockdown of c‐Kit was confirmed using immunoblotting.

### 2.5 Flow cytometry analysis

Adherent cells were detached using a cell dissociation solution (Merck Millipore) and washed with PBS (pH 7.2). The cells were blocked with blocking buffer (1× PBS containing 5% BSA) for 1 h on ice and centrifuged at 1000 ***g*** for 3 min. Next, the cells were incubated with the indicated concentrations of NN2101 diluted in FACS buffer (1× PBS containing 2% BSA) on ice for 1 h. The cells were then washed thrice with FACS buffer and incubated with fluorescein isothiocyanate (FITC)‐conjugated anti‐human IgG (0.5 µg·mL^−1^; Invitrogen) on ice for 1 h. Finally, the cells were washed thrice with FACS buffer and the fluorescent signals were analyzed at 488 nm using CyFlow^®^ Cube 6 (PARTEC, Münster, Germany).

### 2.6 Effector function assay

The effector function assay was performed as described previously [41]. To perform the CDC assay, the cells were seeded in a black 96‐well cell culture microplate (Greiner Bio‐One, Alphen aan den Rijn, Netherlands) and incubated with NN2101. The cells were then treated with 20% (v/v) human serum complement (Quidel, San Diego, CA, USA) and incubated in a humidified CO_2_ incubator. Next, the cells were stained with Calcein AM (Invitrogen), propidium iodide (Sigma Aldrich, St. Louis, MO, USA), and Hoechst 33342 (Invitrogen). The stained cells were counted using a Celigo Imaging Cytometer (Nexcelom Bioscience, Lawrence, KS, USA).

The ADCC assay was performed using the ADCC reporter bioassay, core kit (Promega, Madison, WI, USA). The cells were seeded in a 96‐well Cell Culture Microplate White (Greiner Bio‐One) and treated with NN2101 at RT. *Jurkat* cells stably expressing the FcγRIIIa receptor (V158 variant) and an NFAT response element driving the expression of firefly luciferase were used as effector cells (Promega). The *Jurkat* cells (6 × 10^4^ cells/well) were co‐cultured with the target cells (effector/target ratio = 6 : 1) for 6 h in a humidified CO_2_ incubator. Activation of effector cells was determined by quantifying the luciferase activity using GloMax reagent (Promega), following the manufacturer's instructions.

### 2.7 Cytotoxicity assay

The following cells were seeded into a 96‐well plate and cultured in their respective culture medium containing FBS in a humidified 5% CO_2_ for 12 h: GIST‐T1 (5 × 10^3^ cells/well), GIST‐430 (5 × 10^3^ cells/well), GIST‐430/654 (5 × 10^3^ cells/well), GIST‐48 (8 × 10^3^ cells/well), NCI‐H526 (7 × 10^3^ cells/well), NCI‐H889 (7 × 10^3^ cells/well), NCI‐H1048 (5 × 10^3^ cells/well), NCI‐H2170 (7 × 10^3^ cells/well), TF‐1 (5 × 10^3^ cells/well), Kasumi‐1 (2 × 10^4^ cells/well), HMC1.2 (5 × 10^3^ cells/well), SK‐OV‐3 (1.5 × 10^3^ cells/well), CAOV‐3 (5 × 10^3^ cells/well), OVCAR‐3 (5 × 10^3^ cells/well), MDA‐MB‐468 (4 × 10^3^ cells/well), MDA‐MB‐453 (5 × 10^3^ cells/well), HUVECs (4 × 10^3^ cells/well), MS‐1 (4 × 10^3^ cells/well), and COS7 (5 × 10^3^ cells/well). The cells were then treated with the indicated concentrations of SMCC‐DM1, NN2101, or NN2101‐DM1 for 3–5 days and incubated with Hoechst 33342 (10 µm; Thermo Fisher Scientific) or Calcein AM (1.2 µg·mL^−1^; Invitrogen) in a humidified 5% CO_2_ chamber at 37 °C for 30 min. Live cells were quantified using a Celigo Imaging Cytometer (Nexcelom Bioscience).

### 2.8 Cell cycle and apoptosis assay

Cells were seeded into a 96‐well plate (adherent cells; 0.7–1 × 10^4^ cells/well) or a 6‐well plate (suspension cells; 3 × 10^5^ cells/well) and incubated in a humidified 5% CO_2_ chamber for 24 h. Next, the cells were incubated with vehicle, NN2101 (1 µg·mL^−1^), IgG‐DM1 (1 µg·mL^−1^), or NN2101‐DM1 (1 µg·mL^−1^) for 24 and 48 h. The cells were then fixed with 80% ice‐cold ethanol (adherent cell) at 4 °C for 2 h or 2% paraformaldehyde (suspension cell) at 4 °C for 10 min and washed twice with cold PBS. Further, the cells were stained with propidium iodide solution (propidium iodide, 50 µg·mL^−1^; RNase A, 0.1 mg·mL^−1^; Triton X‐100, 0.05%) at 37 °C for 60 min and analyzed using a Celigo Imaging Cytometer (Nexcelom Bioscience).

For apoptosis assays, the cells (5 × 10^3^–7 × 10^3^/well) were seeded into 96‐well plates and incubated in a humidified CO_2_ chamber for 16 h. The cells were incubated with vehicle, NN2101 (1 µg·mL^−1^), IgG‐DM1 (1 µg·mL^−1^), or NN2101‐DM1 (1 µg·mL^−1^) for 72 h. Next, the cells were stained with caspase 3/7 reagent (2 µm; Nexcelom Bioscience) and Hoechst 33342 (10 µm; Thermo Fisher Scientific) and analyzed using a Celigo Imaging Cytometer.

### 2.9 Internalization assay

To perform the internalization assay using FACS, the cells were incubated with cycloheximide (75 µg·mL^−1^) to prevent the transport of *de novo* synthesized c‐Kit to the cytoplasmic membrane. The cells were blocked with human BD Fc block™ (BD Biosciences, San Diego, CA, USA), to inhibit the Fc receptor‐mediated internalization of antibody, and 5% BSA (CellNest, Gyeonggi‐do, Korea) at RT for 10 min. Next, the cells were washed with PBS and incubated in the presence or absence of NN2101 (1 µg·mL^−1^) at 4 or 37 °C for 1–4 h. The cells were then incubated with FITC‐conjugated anti‐human IgG (0.3 µg·mL^−1^; Invitrogen) for 1 h, washed thrice with PBS, and analyzed using CyFlow^®^ Cube 6 (PARTEC).

To perform confocal microscopy analysis, NN2101 was labeled with phycoerythrin (PE; Abcam, Cambridge, UK), following the manufacturer's instructions. The cells (7 × 10^4^ cells/well) were seeded in Lab‐Tek 4‐well chamber slides (Nunc, Roskilde, Denmark) and cultured in a humidified 5% CO_2_ chamber for 1 or 2 days. Next, the cells were blocked with a mixture of human BD Fc block™ (25 µg·mL^−1^) and 3% BSA for 10 min, followed by incubation with PE‐conjugated NN2101 (10 µg·mL^−1^) at 4 °C for 1 h. The cells were then washed with Dulbecco's PBS (DPBS) containing 1% BSA and cultured in their respective culture media for the indicated time at 37 °C in a humidified 5% CO_2_ chamber. Further, the cells were fixed with 4% paraformaldehyde at RT for 10 min, washed twice with PBS, and permeabilized with 0.1% Triton X‐100 at RT for 15 min. After washing twice with PBS, the cells were blocked with 3% BSA at RT for 1 h and incubated with allophycocyanin (APC)‐conjugated anti‐human CD107a (1 : 50; BD Pharmingen, San Diego, CA, USA) and 4′,6‐diamidino‐2‐phenylindole (100 nm) at RT for 1 h. The cells were washed thrice with DPBS containing 1% BSA and mounted with gel mounting solution (Biomeda, Foster City, CA, USA). The fluorescence images were captured using a confocal microscope (Nikon A1, Tokyo, Japan) and analyzed using nis‐Elements microscope imaging software (Nikon).

### 2.10 Generation of ADC

Human IgG1 (Sino Biological) and NN2101 were dialyzed against conjugation buffer (100 mm sodium phosphate and 150 mm NaCl; pH 7.2) and conjugated with SMCC‐DM1 (MedChemExpress, Monmouth Junction, NJ, USA) at RT with a molar ratio of 1 : 4 for 1.5 h. Fractionation was performed using Superdex™ 200 (GE Healthcare, Wauwatosa, WI, USA) to remove free SMCC‐DM1 and aggregated NN2101‐DM1. The antibody fractions were pooled and dialyzed against storage buffer (10 mm sodium succinate, 0.05% polysorbate 20, and 6% sucrose; pH 5.0). The optical density (OD) at 252 nm was examined to determine conjugation. DAR was calculated using liquid chromatography‐tandem mass spectrometry (LC‐MS) analysis. NN2101‐DM1 was treated with PNGaseF for deglycosylation. The LC‐MS analysis was performed using a Waters Acquity UPLC I‐class system under the following conditions: column, Thermo MAbPac™ RP column (2.1 × 5 mm); mobile phase, 0.1% formic acid in water (eluent A) and 0.1% formic acid in acetonitrile (eluent B); mode, gradient mode. The gradient conditions were as follows: 0–2 min, 25% eluent B; 2–10 min, linear increase to 45% eluent B at a flow rate of 0.2 mL·min^−1^. A Thermo LTQ Orbitrap mass spectrometer (Thermo Fisher Scientific) was used to obtain electrospray ionization (ESI) mass spectra (*m/z* 400–4000) at a mass resolution of 120 000. The obtained ESI mass spectra were deconvoluted using protein deconvolution software (Thermo Fisher Scientific). DAR was calculated using the deconvoluted spectra.

### 2.11 Serum stability assay

NN2101‐DM1 (100 µg) was incubated with low IgG FBS (Promega) at 37 °C in a humidified 5% CO_2_ chamber. At the indicated times, NN2101‐DM1 was precipitated with protein A/G agarose (Santa Cruz Biotechnology) and washed thrice with PBS. NN2101‐DM1 was eluted with 0.1 m glycine (pH 3.0) and neutralized with 10% volume of 1 m Tris‐HCl (pH 8.5). Next, NN2101‐DM1 was analyzed at an OD of 252 nm and calibrated at an OD of 280 nm. The calibrated values were subtracted from the OD of NN2101 and presented as relative values.

### 2.12 *In vivo* experiment

The animal studies were approved by the Ajou University Animal Care and Use Committee (IACUC 2019‐0005, IACUC 2019‐0033). The experiment conformed to the Guide for the Care and Use of Laboratory Animals published by the United States National Institutes of Health. All experiments were performed according to the relevant guidelines and regulations. The animal room had a controlled 12/12‐h light/dark cycle (lights on at 06:00 AM), temperature (20–26 °C), and relative humidity (50 ± 10%). For xenograft assays, mice were anesthetized with isoflurane (Hana Pharm Co., Seoul, Korea). Then, GIST‐T1 (3 × 10^6^ cells/100 µL/mouse), HMC1.2 (1 × 10^6^ cells/100 µL/mouse), NCI‐H526 (2 × 10^6^ cells/100 µL/mouse), Kasumi‐1 (3 × 10^6^ cells/100 µL/mouse), and MDA‐MB‐468 (1.5 × 10^6^ cells/100 µL/mouse) cells in 50% Matrigel (Corning, Corning, NY, USA) were subcutaneously transplanted into 5‐week‐old female C.B‐17 severe combined immunodeficiency (SCID) mice (Orientbio, Seongnam, Korea). GIST‐430/654 (3 × 10^6^/100 µL/mouse) cells were transplanted into 5‐week‐old female NOG mice (Koatech, Pyeongtaek, Korea). Mice with established tumors were randomized into treatment groups for the efficacy study. *In vivo* efficacy studies were initiated when the volume of the tumors reached ~ 200 mm^3^. Imatinib (Santa Cruz Biotechnology) was formulated in distilled water for oral administration. The mice were intravenously administered NN2101, IgG‐DM1, or NN2101‐DM1 three times at the indicated concentrations. Carboplatin (Sigma Aldrich) was prepared in PBS. The mice were intraperitoneally administered carboplatin solution (60 mg·kg^−1^·day^−1^) on days 1 and 11. Etoposide (Sigma‐Aldrich) was prepared in dimethyl sulfoxide, and the solution was diluted in PBS just before administration. The mice were intraperitoneally administered etoposide for 2 cycles (3 mg·kg^−1^·day^−1^; days 1–5 and days 11–15). The tumor volumes were measured twice weekly as follows: volume = (4/3) × π × (length/2) × (width/2) × (depth/2) [45]. Tumor growth inhibition (TGI) rate was calculated as follows: TGI = [1 − (relative tumor volume (RTV) in the treated group)/(RTV in the control group) × 100 (%)]. RTV = (tumor volume on measured day)/(tumor volume on day 0). After the end of the experiment, mice were euthanized using CO_2_.

### 2.13 Statistical analysis

All statistical analyses were performed using graphpad prism 5.0 software (La Jolla, CA, USA). The data are expressed as mean ± standard error of mean. The means were compared using an unpaired Student's two‐sided *t*‐test or one‐way analysis of variance, followed by Tukey's *post hoc* test. The differences were considered significant at *P* < 0.05.

## 3 Results

### 3.1 NN2101 inhibits c‐Kit signaling in various cancer cell lines

**Fig. 1:**
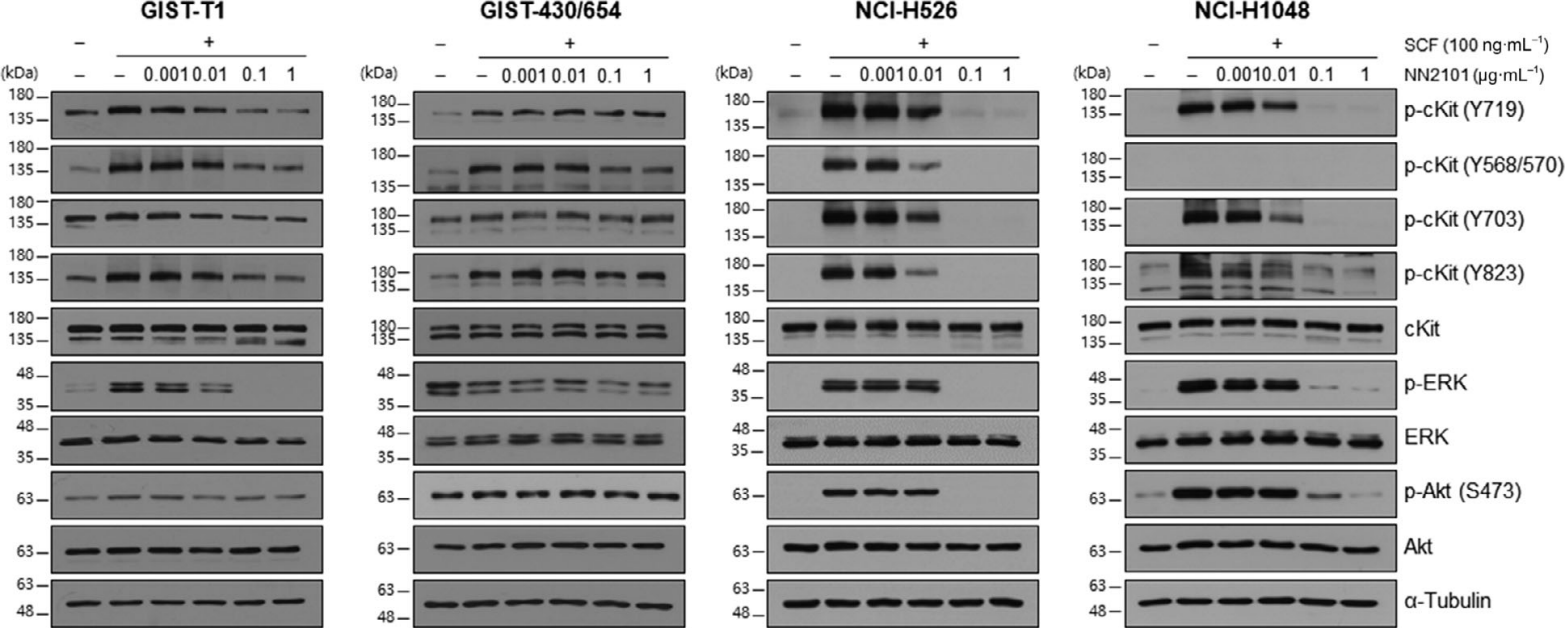
NN2101 inhibits SCF‐mediated signal transduction *in vitro*. Cancer cell lines were serum‐starved for 6 h, followed by pretreatment with NN2101 at the indicated concentration for 1 h and stimulation with human SCF for 5–10 min. Cell lysates were prepared and subjected to western blotting to examine the phosphorylation status of c‐Kit, ERK, and Akt. NN2101 dose‐dependently inhibited the phosphorylation of c‐Kit at various tyrosine residues. However, NN2101 partially inhibited the phosphorylation of c‐Kit in the GIST‐430/654 cells, which can be attributed to the presence of heterozygous c‐Kit genotypes (wild‐type and V560‐L576; del/V654A). Tubulin was used as a loading control. All experiments were independently repeated at least three times.

NN2101 was reported to inhibit the SCF‐dependent activation of c‐Kit in TF‐1 cells [41]. In this study, the effect of NN2101 on the SCF/c‐Kit signaling pathways in other cancer cell lines was examined. Western blotting was performed to analyze the expression levels of c‐Kit in various cancer cell lines, including those derived from leukemia, mast cell tumor, GIST, ovarian cancer, SCLC, and breast cancer. The expression levels of c‐Kit in leukemia (Kasumi‐1), mast cell tumor (HMC1.2), GIST (GIST‐T1, GIST‐430, GIST‐430/654), and SCLC (NCI‐H526, NCI‐H889) cell lines were higher than those in other cell lines (Fig. S1). COS7, NCI‐H446, NCI‐H2170, MDA‐MB‐468, and MDA‐MB‐453, which were used as c‐Kit‐negative cell lines, did not exhibit c‐Kit expression. Next, the effect of NN2101 on the SCF‐mediated signaling pathway in tumor cell lines was examined. Pretreatment with NN2101 for 1 h dose‐dependently inhibited SCF‐mediated c‐Kit phosphorylation in GIST‐T1, NCI‐H526, NCI‐H1048, SK‐OV‐3, and Kasumi‐1 cells (Fig. 1 and Fig. S2). The phosphorylation of ERK and Akt was downregulated upon treatment with NN2101 in these cell lines. However, NN2101 partially inhibited or did not inhibit c‐Kit phosphorylation depending on the phosphorylation site of c‐Kit in GIST‐430/654, GIST‐430 cells, and HMC1.2 cells. Meanwhile, NN2101 completely inhibited the phosphorylation of ERK and Akt in GIST‐430 and HMC1.2 cells but not in GIST‐430/654 cells (Fig. 1 and Fig. S2).

### 3.2 Internalization of NN2101 after binding with c‐Kit

**Fig. 2:**
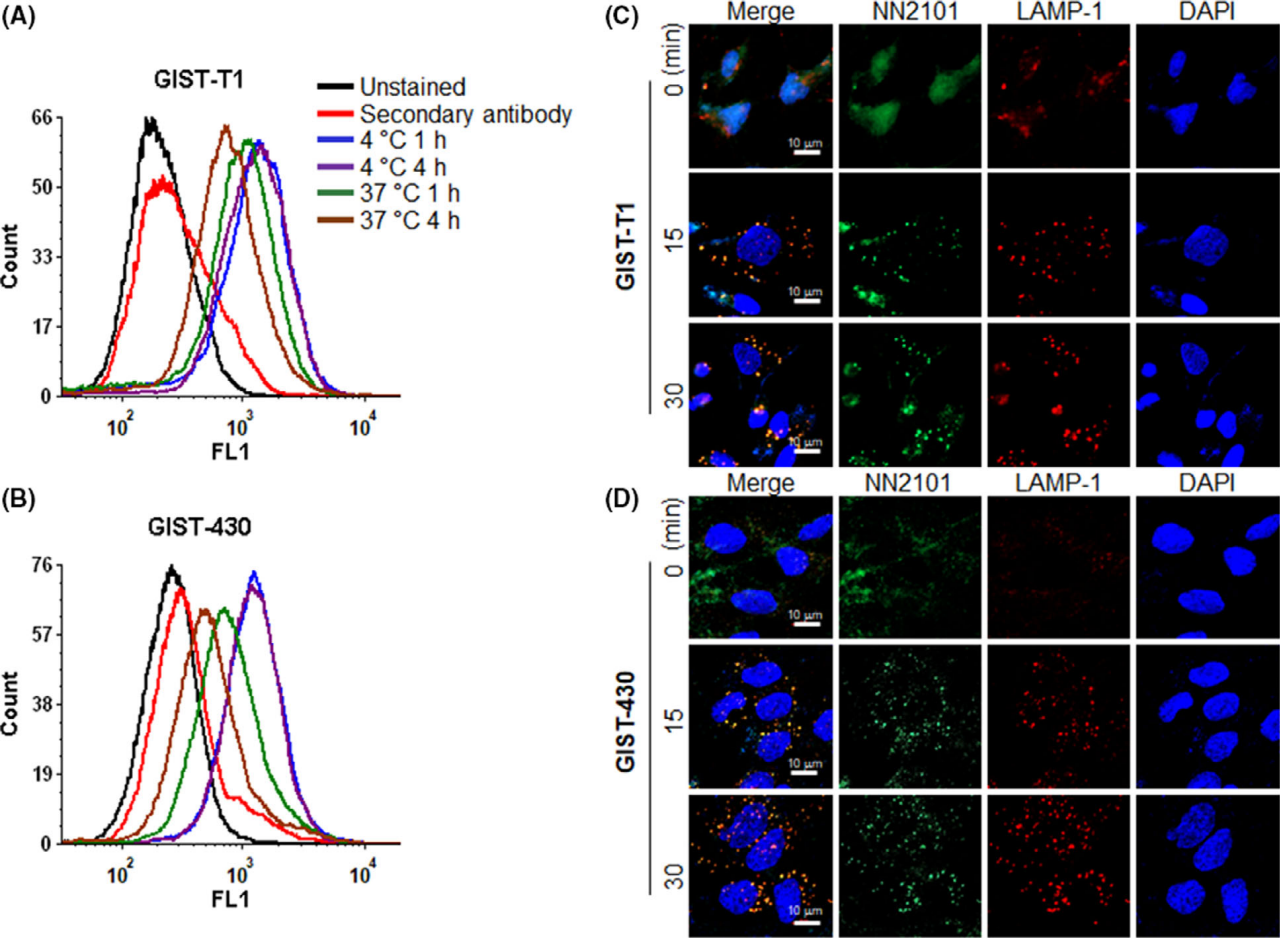
NN2101 is internalized into cancer cells. (A, B) GIST cells were incubated with cycloheximide (75 µg·mL^−1^) and blocked with Fc blocker for 10 min to inhibit the Fc receptor‐mediated internalization. The cells were incubated in the presence or absence of NN2101 (1 µg·mL^−1^) at 4 or 37 °C for 1–4 h and subjected to flow cytometry. The fluorescent signal of the NN2101/c‐Kit complex on the cell surface decreased after incubation at 37 °C. (C, D) GIST cancer cells were blocked with Fc blocker for 10 min and treated with phycoerythrin‐conjugated NN2101 (10 µg·mL^−1^) at 4 °C for 1 h. The cells were washed and incubated at 37 °C for the indicated time. Next, the cells were fixed with 4% paraformaldehyde at RT for 10 min. After washing, the cells were permeabilized with 0.1% Triton X‐100 at RT for 15 min and stained with allophycocyanin‐conjugated anti‐human 107a (LAMP‐1) antibody and 4′,6‐diamidino‐2‐phenylindole (100 nm) at RT for 1 h. The fluorescence images were captured under a confocal microscope. NN2101 co‐localized with LAMP‐1 (a lysosomal marker) after 15 min of incubation (yellow signal). All experiments were independently repeated at least three times.

NN2101 did not completely inhibit c‐Kit phosphorylation in the imatinib‐resistant GIST‐430/654 and HMC1.2 cells, which limits the application of NN2101 as an anti‐cancer agent. To examine the potential use of NN2101 in ADC, the binding of NN2101 to cells was examined using flow cytometry. Additionally, the internalization of c‐Kit was examined by confocal microscopy and flow cytometry. Flow cytometric analyses revealed that NN2101 dose‐dependently bound to various cancer cells (Fig. S3). To further confirm the specific binding of NN2101, knockdown experiments were performed using siRNA. The siRNA‐mediated c‐Kit downregulation was confirmed by western blotting. The knockdown of c‐Kit decreased the binding of NN2101 to cancer cells (Fig. S4). Next, flow cytometric analysis revealed that NN2101 was time‐dependently cleared from the cell surface (Fig. 2A,B and Fig. S5). Confocal microscopy analysis with the lysosomal marker LAMP‐1 revealed that the NN2101/c‐Kit complex was rapidly trafficked to the lysosome (Fig. 2C,D). These findings indicate that NN2101 is efficiently internalized. As the NN2101/c‐Kit complex was localized to the lysosome, the effect of NN2101 on the degradation of c‐Kit protein was examined. Western blotting analysis revealed that treatment with NN2101 downregulated the expression of c‐Kit in various cancer cells (Fig. S6), which was consistent with the results of a previous study [37].

### 3.3 c‐Kit ADCs exerted cytotoxic effects against various cancer cell lines

**Fig. 3:**
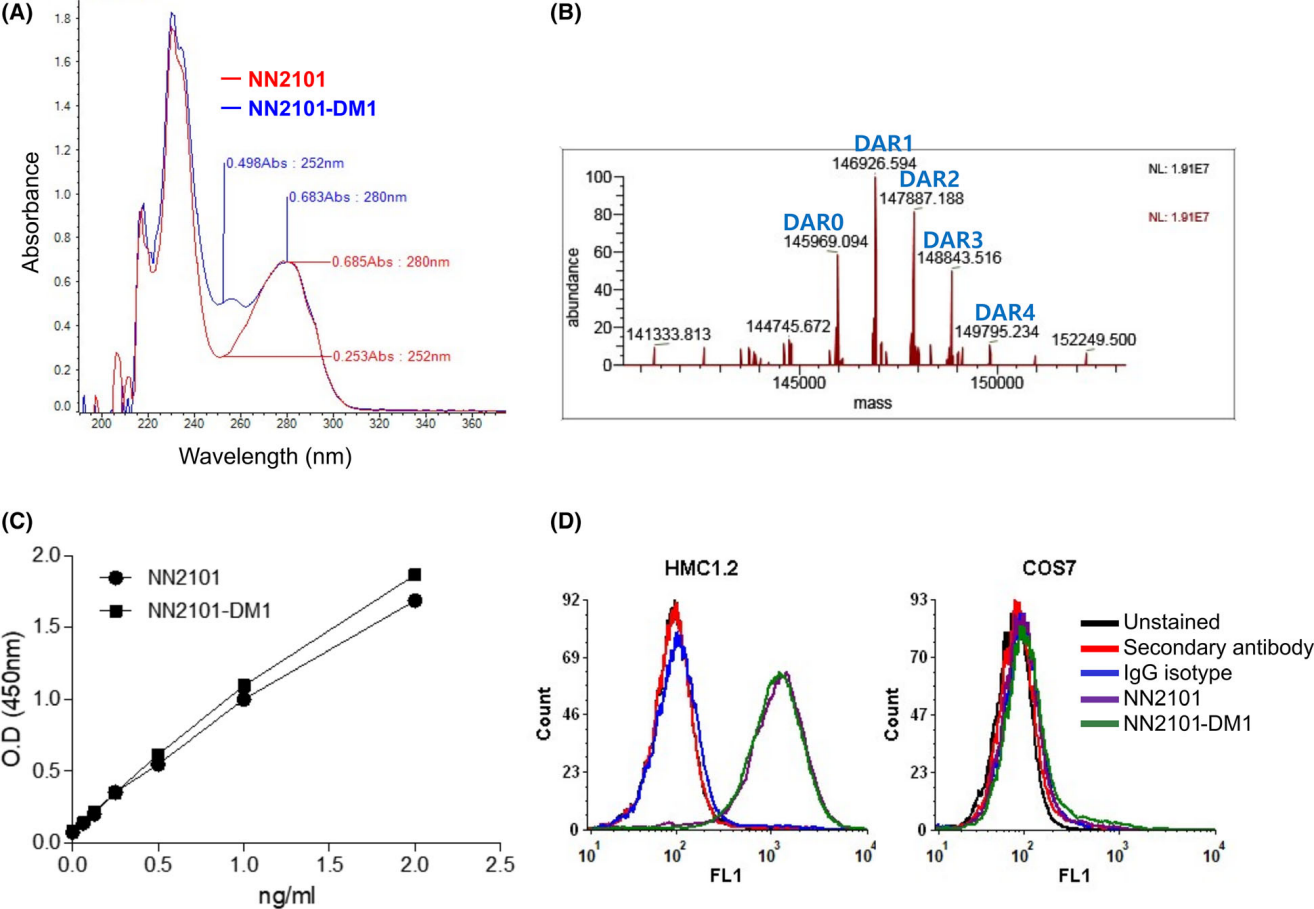
Characterization of NN2101‐DM1. (A) Optical absorbance at 252 nm of NN2101‐DM1 was compared with that of NN2101. (B) Drug‐antibody ratio was visualized as DAR and determined as described in the Materials and Methods section. The binding affinity of NN2101 and NN2101‐DM1 to c‐Kit was compared using ELISA (C) and flow cytometry (D). The c‐Kit binding affinities of NN2101 and NN2101‐DM1 were similar. COS7 cells were used to examine the non‐specific binding of c‐Kit ADC to c‐Kit negative cells. The cells were treated with 10 µg·mL^−1^ of NN2101 or NN2101‐DM1 and subjected to flow cytometry analysis. All experiments were independently repeated at least three times.

**Fig. 4:**
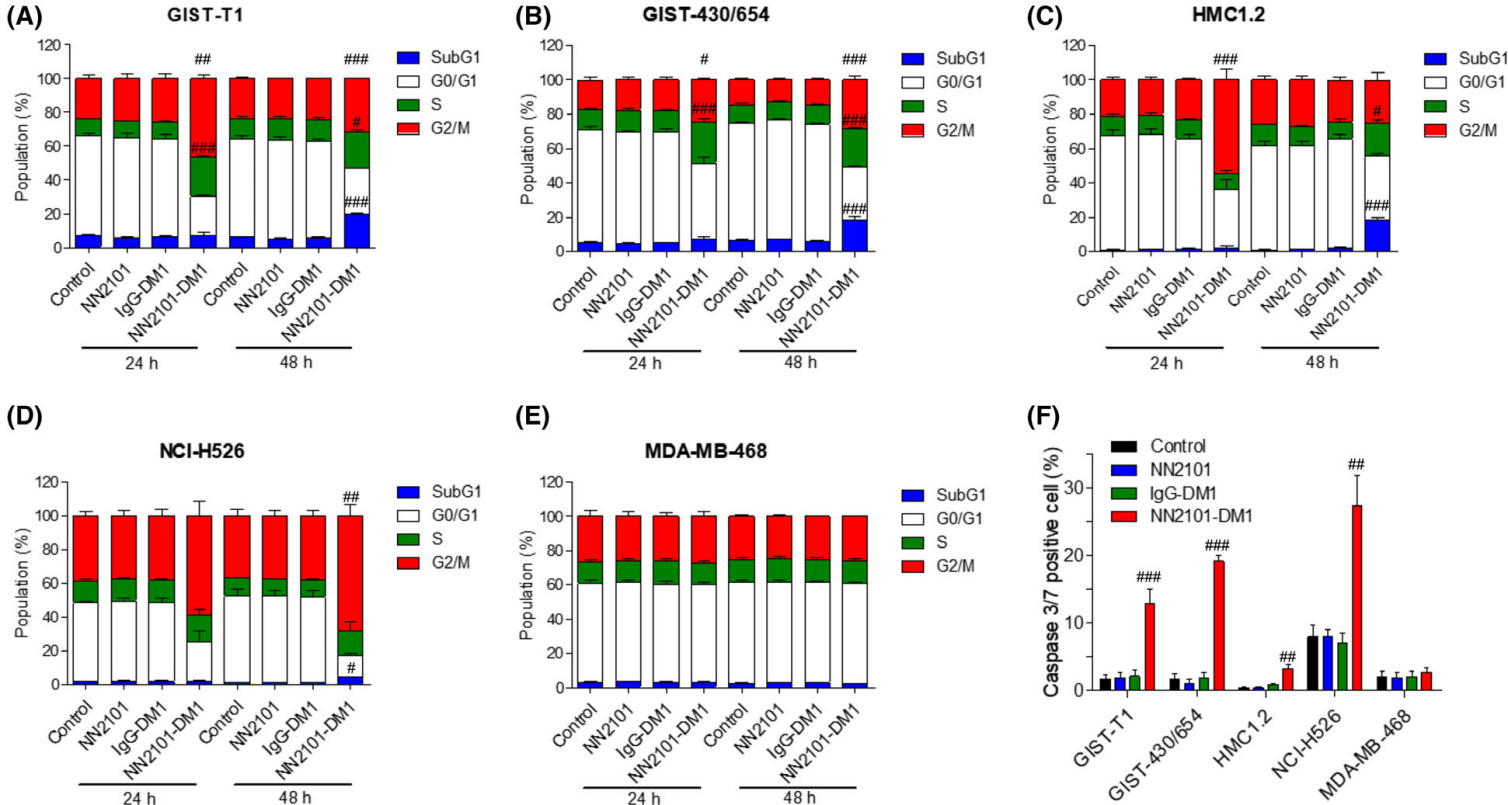
NN2101‐DM1 induces cell cycle arrest and apoptosis. (A–E) Cells were seeded into 96‐well plates and incubated with NN2101 (1 µg·mL^−1^), IgG‐DM1 (1 µg·mL^−1^), or NN2101‐DM1 (1 µg·mL^−1^) for 24 and 48 h. Next, the cells were fixed with 80% ice‐cold ethanol at 4 °C for 2 h and washed with cold PBS. The cells were then stained with propidium iodide and analyzed using a Celigo Imaging Cytometer (#, ##, and ### vs. their respective corresponding control, NN2101, and IgG‐DM1). NN2101‐DM1 increased the proportion of cells at the G2/M and S phases at 24 h. Additionally, NN2101‐DM1 increased the proportion of cells at the subG1 phase at 48 h. The results represent mean ± standard error of mean (SEM) from at least three independent experiments. (F) For apoptosis assays, cells were seeded into 96‐well plates and incubated with vehicle, NN2101 (1 µg·mL^−1^), IgG‐DM1 (1 µg·mL^−1^), or NN2101‐DM1 (1 µg·mL^−1^) for 72 h. The cells were stained with caspase 3/7 reagent (2 µm) and Hoechst 33342 (10 µm) and analyzed using a Celigo Imaging Cytometer (## and ### vs. their respective corresponding control, NN2101, and IgG‐DM1). NN2101‐DM1 increased the proportion of caspase 3/7‐positive cells, which indicated that it increased apoptosis. The results represent mean ± SEM from at least three independent experiments. The means were compared using an one‐way analysis of variance, followed by Tukey's *post hoc* test. ^#^ *P* < 0.05, ^##^ *P* < 0.01, and ^###^ *P* < 0.001.

Next, NN2101‐DM1 was generated using SMCC‐DM1, which comprises an uncleavable linker and a microtubule inhibitor. As DM1 absorbs ultraviolet light at 252 nm [46], the absorbance of naked antibody (NN2101) and ADC (NN2101‐DM1) at 252 nm was comparatively analyzed. The absorbance of NN2101‐DM1 was higher than that of NN2101 at 252 nm (Fig. 3A). The DAR of NN2101‐DM1 was approximately 1.51 (Fig. 3B). The enzyme‐linked immunosorbent assay (ELISA) and flow cytometric analyses revealed that the binding affinities of NN2101 and NN2101‐DM1 to c‐Kit were similar (Fig. 3C,D). Surface plasmon resonance analysis revealed that the binding affinity of NN2101‐DM1 to human c‐Kit ranged from 8 × 10^−12^ m to 24 × 10^−12^ m, which was consistent with the results of our previous study [41]. DM1 induces cell cycle arrest at the G2/M phase by inhibiting the assembly of microtubules and consequently promotes apoptosis. Hence, the effect of NN2101‐DM1 on the cell cycle was analyzed. The proportion of cells at the S and G2/M phases at 24 h in the NN2101‐DM1‐treated imatinib‐sensitive and imatinib‐resistant cancer cell lines was significantly higher than that in the control, NN2101‐treated, and IgG‐DM1‐treated imatinib‐sensitive and imatinib‐resistant cancer cell lines. However, NN2101‐DM1 did not affect the proportion of cells at the S and G2/M phases in the c‐Kit‐negative cell lines (Fig. 4A–E). Additionally, NN2101‐DM1 significantly increased the proportion of cells at the subG1, S, and G2/M phases at 48 h in the c‐Kit‐positive cancer cell lines but not in the c‐Kit‐negative cell lines. This indicated that NN2101‐DM1 promotes apoptosis in the c‐Kit‐positive cancer cell lines. To further confirm these results, the levels of active caspase 3/7, which are apoptosis markers, were analyzed. Treatment with NN2101‐DM1 significantly increased the proportion of caspase 3/7‐positive cells in the c‐Kit‐positive cancer cell lines but not in the c‐Kit‐negative cells (Fig. 4F). These results suggest that NN2101‐DM1, but not NN2101, induces apoptosis irrespective of imatinib resistance status in the c‐Kit‐positive cancer cells. Studies to confirm whether the cell lines used in this study are sensitive or resistant to imatinib were conducted *in vitro*, and results for the cell lines described here were shown to be consistent with previous other results (results not shown) [47, 48, 49, 50, 51].

**Fig. 5:**
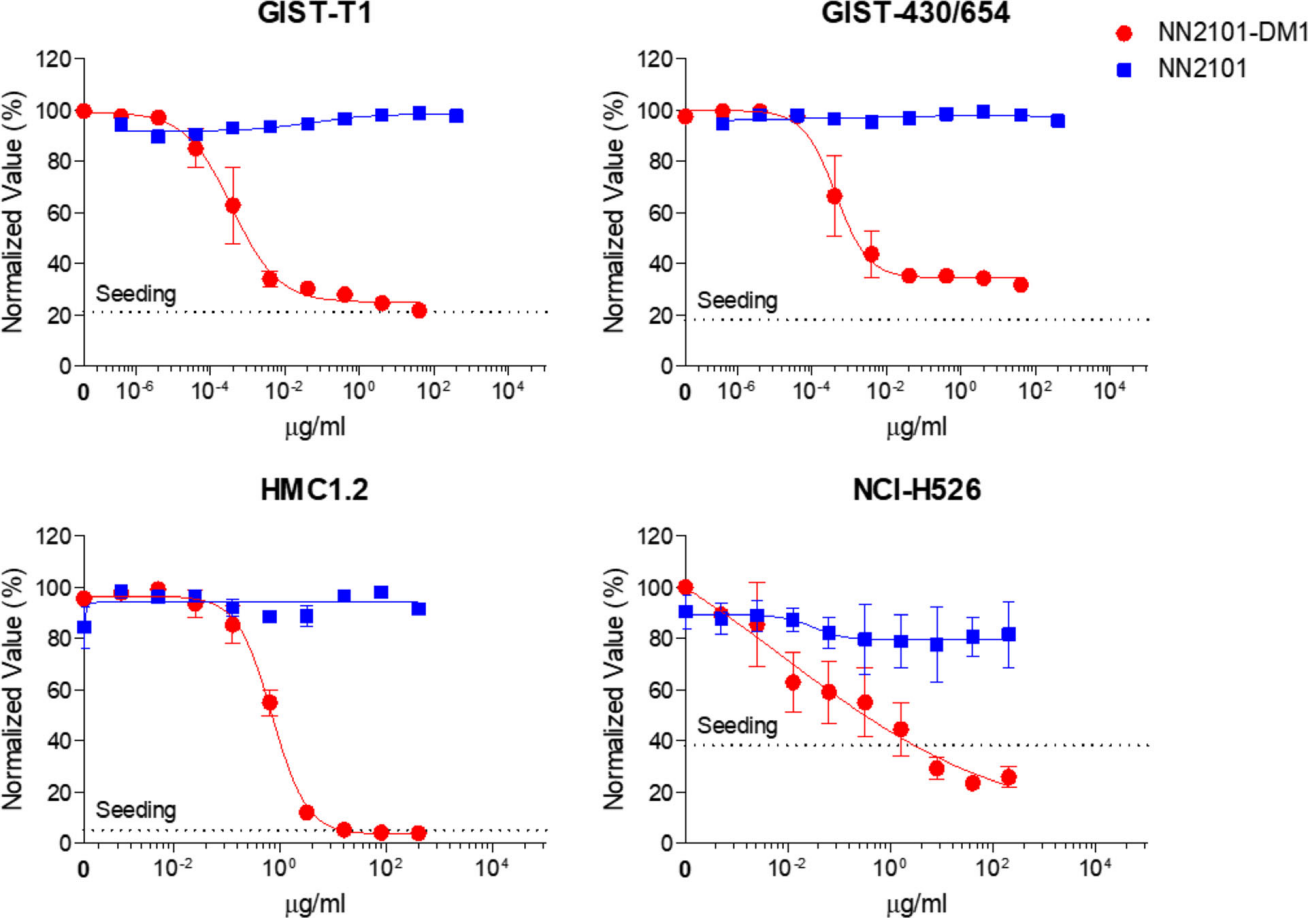
NN2101 exerts cytotoxic effects against cancer cells *in vitro*. Cells were seeded into 96‐well plates and incubated with diluted doses of NN2101 or NN2101‐DM1 for 3–5 days. Live cells were stained with Hoechst 33342 (10 µm) or Calcein AM (1.2 µg·mL^−1^) at 37 °C for 30 min and quantitated using a Celigo Imaging Cytometer. NN2101‐DM1 dose‐dependently decreased cell viability. The results represent mean ± standard error of mean from at least three independent experiments.

The activity of NN2101‐DM1 was examined in various cancer cell lines. The cytotoxicity activities of NN2101‐DM1 against leukemia cells (TF‐1, Kasumi‐1, and HMC1.2), GIST (GIST‐T1, GIST‐430, GIST‐430/654, and GIST‐48), SCLC cells (NCI‐H526, NCI‐H889, and NCI‐H1048), ovarian cancer cells (SK‐OV‐3, CAOV‐3, and OVCAR‐3), and endothelial cells (HUVEC and MS‐1) were examined. The c‐Kit‐negative cell lines (MDA‐MB‐453, MDA‐MB‐468, NCI‐H2170, and COS7) served as negative controls. NN2101‐DM1 exhibited *in vitro* cytotoxic activities against HMC1.2, GIST‐T1, GIST‐430, GIST‐430/654, NCI‐H526, and NCI‐H1048 cells with the half‐maximal inhibitory concentration (IC_50_) values in the nanomolar to picomolar range. The *in vitro* cytotoxic activities of NN2101‐DM1 against c‐Kit‐positive cell lines were 4‐fold to > 10 000‐fold higher than that against c‐Kit‐negative cell lines (Fig. 5 and Fig. S7; Table 1).

**Table 1:** Half‐maximal inhibitory concentration values of tested materials. N.D., not determined up to 1775 nm; N/A, not available; N/E, not effective; N/T, not tested.

c‐Kit expression	Tissue type	Cell	NN2101‐DM1 (µg·mL^−1^)	SMCC‐DM1 (nm)	c‐Kit mutation	Imatinib sensitivity	Reference
c‐Kit‐positive	Leukemia	TF‐1	7.384	21.96	Wild‐type	Resistant	
		Kasumi‐1	5.473	58.46	N822K	Intermediate resistant	[71]
		HMC1.2	0.7673	13.47	V560G/D816V	Resistant	[72]
	GIST	GIST‐T1	0.0003764	6.154	V560‐V578;del	Sensitive	[73]
		GIST‐430	0.002018	78.03	V560‐L576;del	Sensitive	[74]
		GIST‐430/654	0.000433	12.62	V560‐L576;del/ V654A	Resistant	[74]
		GIST‐48	17.49	29.39	V560D/D820A	Resistant	[74]
	SCLC	NCI‐H1048	1.18	2.277	N/A	N/E	
		NCI‐H526	0.008068	7.713	N/A	N/E	
		NCI‐H889	12.47	N.D.	N/A	N/E	
	Ovarian cancer	SK‐OV‐3	4.304	42.47	N/A	N/T	[75]
		Caov‐3^a^	2.524	41.94	N/A	N/T	[75]
		OVCAR‐3^a^	4.03	34.23	N/A	N/T	[76]
	Endothelial cell	HUVEC	11.95	155.2	N/A	N/E	[77]
		MS‐1	17.63	118.6	N/A	N/T	
c‐Kit‐negative	SCLC	NCI‐H2170	3.707	17.60	N/A	N/E	
	Breast cancer	MDA‐MB‐453	2.991	9.702	N/A	N/E	
		MDA‐MB‐468	2.541	21.67	N/A	N/E	
	Kidney	COS7	11.84	37.04	N/A	N/E	

^a^ Extremely low expression of c‐Kit and negligible as determined using western blotting.

### 3.4 NN2101‐DM1 exhibits antitumor activity in mouse xenograft models

**Fig. 6:**
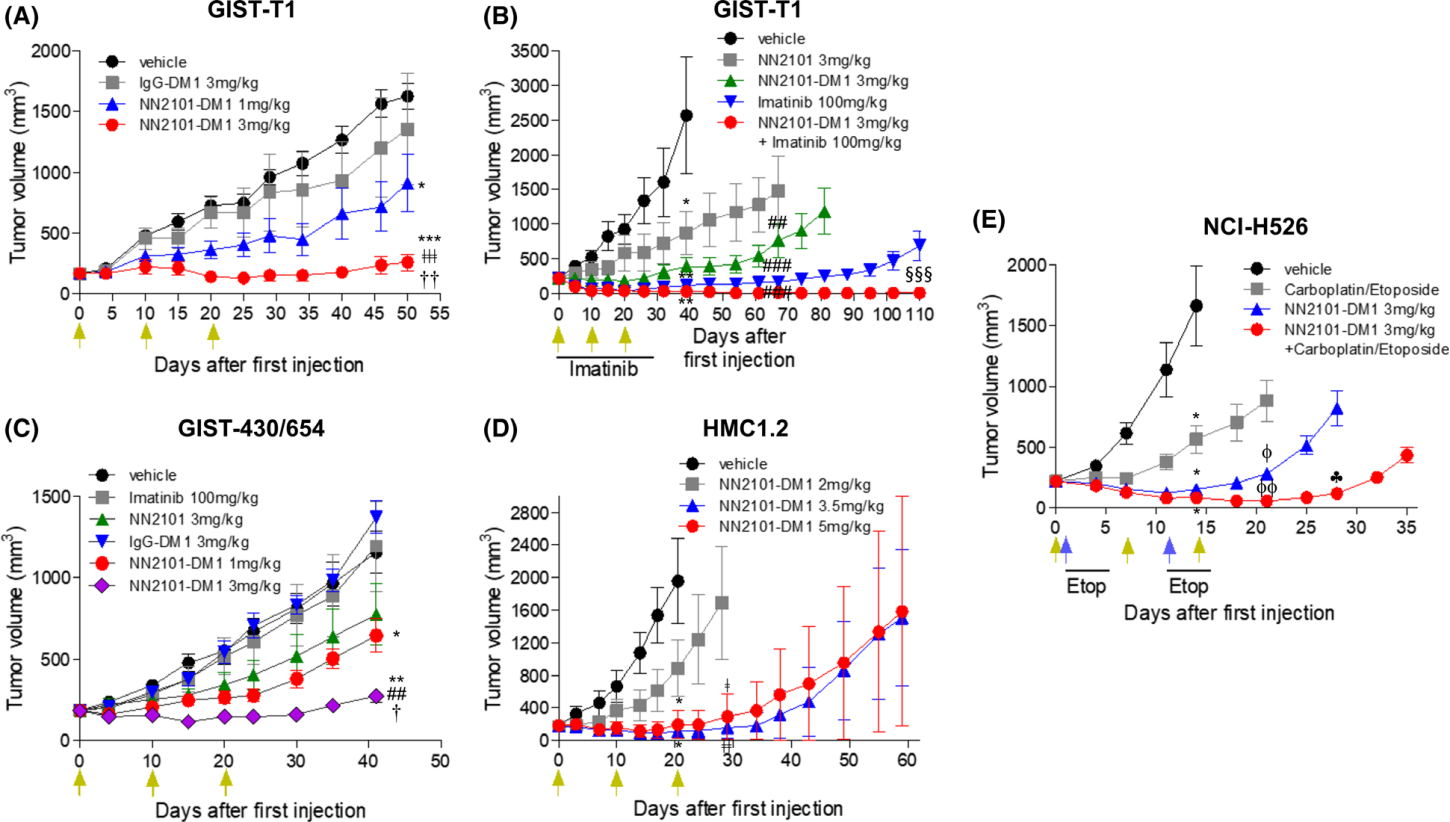
Antitumor activity of NN2101‐DM1 in *in vivo* xenograft mouse models. Cancer cells were implanted into immunodeficient mice. Mice with established tumors were randomized into different treatment groups when the volume of the tumors reached ~ 200 mm^3^ (*n* = 6 or 7). Animals were intravenously administered vehicle, NN2101, IgG‐DM1, or NN2101‐DM1. Additionally, the animals were orally administered imatinib (100 mg·kg^−1^) or a combination of NN2101‐DM1 and imatinib once daily or intraperitoneally administered carboplatin (60 mg·kg^−1^·day^−1^) on days 1 and 11 and etoposide (3 mg·kg^−1^·day^−1^ on days 1–5 and days 11–15 or a combination of NN2101‐DM1 and carboplatin/etoposide. Tumor volumes were plotted against days after drug administration. Green arrows indicate the administration of vehicle, NN2101, IgG‐DM1, or NN2101‐DM1, while the blue arrows indicate the administration of carboplatin. (*, **, and *** vs. their respective corresponding vehicle; ## and ### vs. their respective corresponding NN2101 (3 mg·kg^−1^); ǂ and ǂǂ vs. NN2101‐DM1 (2 mg·kg^−1^); ǂǂǂ vs. IgG‐DM1; † and †† vs. NN2101‐DM1 (1 mg·kg^−1^); §§§ vs. imatinib; ϕ and ϕϕ vs. carboplatin/etoposide; ♣ vs. NN2101‐DM1 (3 mg·kg^−1^). The results represent the mean ± SEM of at least three independent experiments. The means were compared using an unpaired Student's two‐sided *t*‐test. *P < 0.05, **P < 0.01, ***P < 0.001, ^##^ *P* < 0.01, ^###^ *P* < 0.001, ^ǂ^ *P* < 0.05, ^ǂǂ^ *P* < 0.01, ^ǂǂǂ^ *P* < 0.001, ^†^ *P* < 0.05, ^††^ *P* < 0.01, ^§§§^ *P* < 0.001, ^ϕ^ *P* < 0.05, ^ϕϕ^ *P* < 0.01, and ^♣^ *P* < 0.05.

Before administration to an *in vivo* xenograft mouse model, the serum stability of NN2101‐DM1 was examined. NN2101‐DM1 was incubated with low IgG FBS for different durations, and the mixture was subjected to immunoprecipitation using protein A/G sepharose. The absorbance of NN2101‐DM1 was compared with that of NN2101 at 252 nm. The relative OD value of NN2101‐DM1 was > 50% even on day 16 (Fig. S8). Additionally, the half‐life of NN2101 was 11 days in rats, which was comparable to that of trastuzumab (results not shown) [52]. Based on these results, the dosing interval was determined to be in the range of 7–10 days depending on the cancer type. The *in vivo* efficacy of NN2101‐DM1 was examined using mouse models xenotransplanted with imatinib‐sensitive GIST‐T1, imatinib‐resistant GIST‐430/654, leukemia (HMC1.2 and Kasumi‐1), and SCLC (NCI‐H526) cell lines. NN2101 (3 mg·kg^−1^) partially suppressed GIST‐T1 tumor growth, while NN2101‐DM1 (3 mg·kg^−1^) promoted tumor stasis for approximately 50 days with subsequent regrowth (Fig. 6A,B). Imatinib suppressed the growth of tumors. However, the growth of tumors was not suppressed after the cessation of imatinib treatment. The combination of NN2101‐DM1 (3 mg·kg^−1^) and imatinib resulted in the complete remission of tumors, with no regrowth even after cessation of imatinib administration for up to 110 days. To examine the therapeutic efficacy of NN2101‐DM1 against imatinib‐resistant cancer cell line, mice bearing GIST‐430/654 tumors were administered various doses of NN2101‐DM1. Imatinib and IgG‐DM1 did not suppress tumor growth. NN2101 partially suppressed tumor growth (Fig. 6C). The TGI rates of NN2101‐DM1 at doses of 1 and 3 mg·kg^−1^ were 44% and 76%, respectively (day 41), when compared with those of vehicle control. In mice bearing HMC1.2 mast cell tumors, the antitumor effects of NN2101‐DM1 at a dose of 5 mg·kg^−1^ were higher than those of vehicle, NN2101, IgG‐DM1, and NN2101‐DM1 (2 mg·kg^−1^) with no tumor regression upon imatinib administration (Fig. S9). The results of an additional experiment revealed that NN2101‐DM1 at a dose of 2 mg·kg^−1^ partially suppressed tumor growth (TGI 54% at day 21). The TGI rates of NN2101‐DM1 at doses of 3.5 and 5 mg·kg^−1^ were 94% and 90%, respectively, at 21 days with subsequent regrowth (Fig. 6D). The analyses of individual mice administered NN2101‐DM1 at doses of 3.5 and 5 mg·kg^−1^ revealed that one mouse from each group exhibited rapid tumor regrowth after the second dose for unknown reasons (Fig. S10). All mice administered NN2101‐DM1 at doses of 3.5 and 5 mg·kg^−1^, except for one mouse in each group, exhibited complete remission until 40 and 45 days, respectively. In mice bearing NCI‐H526 tumors, the TGI rate of NN2101‐DM1 at a dose of 1 mg·kg^−1^ was 45% on day 14. Meanwhile, the TGI rate of NN2101‐DM1 at doses of 3 and 5 mg·kg^−1^ was 95% with subsequent tumor regrowth after 20 days (Fig. S9B). Next, the tumors were treated using a combination of NN2101‐DM1 and carboplatin/etoposide. The TGI rate of carboplatin/etoposide was 66% on day 14, whereas that of NN2101‐DM1 and the combination of NN2101‐DM1 and carboplatin/etoposide was 90% and 95%, respectively (Fig. 6E). On day 21, the TGI rates of NN2101‐DM1 and the combination of NN2101‐DM1 and carboplatin/etoposide were 68% and 94%, respectively, when compared with that of carboplatin/etoposide. This suggested that the combination of NN2101‐DM1 and carboplatin/etoposide exerted potent therapeutic effects by delaying tumor regrowth. In Kasumi‐1 cells, the expression of c‐Kit was upregulated and the NN2101/c‐Kit complex was efficiently internalized (Figs S1 and S5). However, the *in vitro* cytotoxicity and *in vivo* antitumor activity of NN2101‐DM1 against Kasumi‐1 were lower than those of other cancer cell lines (Figs S7 and S9C; Table 1). Additionally, NN2101‐DM1 did not exhibit growth‐inhibitory activity against MDA‐MB‐468 cells, which are c‐Kit‐negative cells, in the xenotransplant mouse model (Fig. S9D). The bodyweight of mice was not affected upon treatment with ADC. In contrast, carboplatin/etoposide treatment decreased the mouse bodyweight, which recovered subsequently (Fig. S11).

## 4 Discussion

In the last two decades, various therapeutics, including imatinib, sunitinib, regorafenib, avapritinib, and ripretinib, have been developed for GIST. However, some patients do not respond to these therapeutics and exhibit tumor recurrence, which can be attributed to the continuous gain of c‐Kit activating mutations [53, 54]. Therefore, an ADC (NN2101‐DM1) was developed in this study to treat various c‐Kit‐positive cancers. NN2101‐DM1 can exert growth‐inhibitory effects against cancers irrespective of their c‐Kit mutational status as the antibody‐c‐Kit complex is internalized and localized to the lysosome. Additionally, c‐Kit‐targeting ADCs can treat cancer cells overexpressing wild‐type or mutant c‐Kit even if the SCF/c‐Kit pathway is not the major pathway mediating the pathogenesis of cancer (as observed in SCLC). Thus, ADC targeting c‐Kit can broaden the therapeutic spectrum.

The binding of SCF to c‐Kit on the cytoplasmic membrane promotes the phosphorylation of wild‐type c‐Kit. The constitutively activated mutant of c‐Kit is phosphorylated in the endoplasmic reticulum and Golgi apparatus, which leads to the induction of SCF‐independent oncogenic signals [55, 56]. This explains the partial inhibition or non‐inhibition of the constitutively activated c‐Kit mutant by the antibody, which was observed in GIST‐430/654 and HMC1.2 cells (Fig. 1 and Fig. S2). NN2101 partially inhibited SCF‐induced Akt and ERK phosphorylation, which is due to the heterozygous genotype of the cancers harboring both wild‐type and mutant c‐Kit [57]. Thus, it is likely that NN2101 inhibits the phosphorylation of only wild‐type c‐Kit located in the cytoplasmic membrane. Although NN2101 inhibits Akt activation, p53 (a downstream target of Akt) is inactivated in various cancers, including SCLC [58, 59, 60]. Thus, these cancers are resistant to apoptosis. Imatinib is the standard therapy for GIST. The probability of acquiring an activating mutation and consequently developing imatinib resistance within 1–2 years of treatment in patients with GIST is > 90% [4], which limits the antitumor efficacy of the antibody. Thus, ADC is a potential therapeutic for cancer (Fig. 6C and Fig. S9A). The *in vitro* IC_50_ values of NN2101‐DM1 were similar against both imatinib‐resistant (GIST‐430/654) and imatinib‐sensitive (GIST‐T1) cells. However, the results of *in vivo* experiments revealed that the efficacy of NN2101‐DM1 against GIST‐430/654 cells was lower than that against GIST‐T1 cells (Fig. 6A–C). Two possibilities can explain this result. First, the viability of GIST‐T1 and GIST‐430/654 cells was 20% and 30%, respectively, after treatment with NN2101‐DM1 even at the saturated treatment concentration (Fig. 5). GIST‐430/654 cells express auto‐activating c‐Kit mutant that induces cell proliferation in the absence of SCF binding. Hence, auto‐activating c‐Kit promotes the relapse of tumors derived from residual GIST‐430/654 cells in the xenograft model after the cessation of NN2101‐DM1 treatment. Second, the C.B‐17 SCID mouse used for GIST‐T1 xenograft experiments harbors functional natural killer (NK) cells. However, since GIST‐430/654 cells did not grow in C.B‐17 SCID mice, NOG mouse that does not have functional NK cell was used in this study as reported in other studies [61]. Some studies have also used the SCID‐beige xenograft model, another NK cell‐depleted mouse model, to examine the efficacy of LOP628 against GIST‐430/654 cells [37]. Thus, the growth‐inhibitory efficacy of NN2101‐DM1 against tumors may be affected by the presence or absence of NK cells in the experimental mouse.

Recently, Abrams *et al*. [37] developed a c‐Kit‐targeting ADC (LOP628) using SMCC‐DM1 and demonstrated its potent antitumor efficacy in preclinical investigations. However, this ADC was associated with hypersensitivity reactions induced by enhanced engagement of IgG with Fc receptor even at low doses in a phase I clinical trial [38]. The effector functions (CDC, ADCC, and antibody‐dependent cellular phagocytosis) of the antibody are critical for its therapeutic efficacy against cancer. As ADC induces immunogenic cell death and exerts cytotoxic effects against cancer cells [62], the antibody effector functions may have minimal roles in the antitumor efficacy. Enhanced effector function of the antibody can cause serious adverse effects due to hypersensitivity reactions. NN2101 did not exhibit CDC and ADCC activities in various cells (Figs S12 and S13) [41], which suggested that NN2101 would have at least a reduced effector function.

Adverse effects can occur either on‐target or off‐target. The on‐target adverse effects (e.g. myelosuppression) induced by c‐Kit‐targeting ADC during cancer treatment must be minimized as c‐Kit plays a crucial role in hematopoiesis. We hypothesized that the application of a DNA‐damaging agent as a payload could induce damage of quiescent or slow dividing c‐Kit‐positive normal cells and consequently increases the on‐target adverse effects. Thus, we selected DM1, a microtubule inhibitor, as a payload. Off‐target adverse effects could be induced by the non‐specific binding of the candidate antibody. Previously, we demonstrated that NN2101 binds to FAS‐associated factor 1 localized in the cytoplasm and nucleus, which suggested that NN2101 does not specifically bind to other extracellular proteins [41]. To further examine the off‐target binding of NN2101, the MDA‐MB‐453 cells, which are c‐Kit‐negative cells, were subjected to immunoprecipitation. The eluted total proteins were subjected to whole‐gel analysis using LC‐MS/MS. Among the cytoplasmic membrane proteins, the isolated NN2101‐binding candidates were CD98 and transferrin receptor protein‐1, which are correlated with tumorigenesis [63, 64]. However, the results of the reconfirmation experiment revealed that NN2101 did not bind to CD98 and transferrin receptor protein‐1 (results not shown), which further suggested that NN2101 specifically binds to c‐Kit.

As SCF binds to c‐Kit with high affinity (1.5 × 10^−9^ m to 3 × 10^−10^ m), a c‐Kit‐targeting antibody with a binding affinity of > 10 pm should be developed to efficiently disrupt the SCF/c‐Kit signaling [65, 66]. The binding affinity of NN2101 to human c‐Kit is reported to be in the picomolar range [41], which can explain the potent antitumor activity of NN2101‐DM1 irrespective of low DAR. The SCF/c‐Kit pathway is the main mediator of hematopoiesis. Hence, NN2101 can induce severe hematologic adverse events when applied as an ADC. However, NN2101‐DM1 did not induce bodyweight loss in mice even though NN2101 is reported to cross‐react with murine c‐Kit (*K*_D_ = 11.5 × 10^−9^ m) [41]. Additionally, the intravenous administration of NN2101‐DM1 at a dose of 40 mg·kg^−1^ did not decrease the bodyweight. Hematological analysis revealed that NN2101‐DM1 (40 mg·kg^−1^) induced significant decrease in the monocyte and eosinophil counts. However, the reduction in the monocyte and eosinophil counts was not clinically significant (results not shown). The IC_50_ value of NN2101‐DM1 against human peripheral blood mononuclear cells was not obtained even after *in vitro* treatment with doses up to 100 µg·mL^−1^ (Fig. S14). This suggested that the adverse hematological events induced by NN2101‐DM1 can be minimal or manageable. However, further studies are needed to examine the safety profile of NN2101‐DM1.

The two isoforms of c‐Kit are GNNK^−^ and GNNK^+^ [67]. The binding affinities of GNNK^−^ and GNNK^+^ to SCF are identical. The c‐Kit GNNK^−^ form induces contact inhibition loss, anchorage‐independent growth, and tumorigenicity by promoting the phosphorylation of ERK and Akt after rapid internalization [67, 68]. Therefore, the GNNK^−^/GNNK^+^ ratio and c‐Kit expression must be examined to predict the response rate of patients before the application of c‐Kit‐targeting ADC for treating cancer. The expression of target molecules in tumor tissues is heterogeneous. Hence, ADC‐mediated clearance of tumor cells exhibiting downregulated expression of such target molecules is challenging, which is a major limitation for achieving complete remission of tumors through ADC. In this study, the cell lines exhibiting downregulated c‐Kit expression (TF‐1, GIST‐48, NCI‐H1048, SK‐OV‐3, CAOV‐3, and OVCAR‐3 cells) were not sensitive to NN2101‐DM1 (Fig. S1 and Table 1). Additionally, the Kasumi‐1 and NCI‐H889 cells were not sensitive to NN2101‐DM1 even though they exhibited upregulated expression of c‐Kit. This may be due to the slow growth kinetics of Kasumi‐1 cells, which limit the efficacy of DM1, a microtubule inhibitor. Therefore, the cell proliferation rate and c‐Kit expression levels of tumor tissues must be considered before the application of DM1 as a payload. In addition, TF‐1 cells exhibit upregulated expression of BCL‐2, an anti‐apoptotic protein [69]. Kasumi‐1 harbors oncogenic fusion of RUNX1‐ETO, which is one of the most common genetic alterations in leukemia and suppresses the expression of Cathepsin G and elastase in the lysosomes [70]. This oncogenic fusion inhibits the release of payload from ADC to induce apoptosis. Therefore, a sufficient efficacious concentration of payload is needed for ADC to induce apoptosis in cancer cells. However, various factors, including downregulated expression of c‐Kit on the cell surface, protease in the lysosomes, upregulated expression of anti‐apoptotic proteins, p53 mutation, and slow growth kinetics of cancer cells limit the therapeutic efficacy of microtubule inhibitors. Hence, the combination of ADC and chemotherapy, immune checkpoint inhibitors, or TKIs should be considered for treating different cancer types. As shown in Fig. 6B,E, the combination of NN2101‐DM1 and imatinib (for GIST) or chemotherapy (for SCLC) will overcome the limitations associated with downregulated expression of c‐Kit in cancer and exert potent therapeutic effects.

## 5 Conclusions

In this study, we developed a novel fully human c‐Kit‐targeting ADC (NN2101‐DM1). NN2101‐DM1, which specifically binds to c‐Kit, was efficiently internalized and targeted to the lysosomes of cancer cells. Additionally, NN2101‐DM1 exhibited *in vitro* growth‐inhibitory efficacy against GIST, SCLC, and SM cells in the nanomolar to picomolar ranges. Some c‐Kit‐positive cells exhibited poor responses to NN2101‐DM1 even though they exhibited upregulated c‐Kit expression and efficiently internalized NN2101‐DM1, which may be due to the decreased degradation of antibody in the lysosomes or upregulated expression of drug‐efflux proteins, such as multi‐drug resistance protein 1. The results of the *in vivo* experiments revealed that the combination of NN2101‐DM1 and imatinib promoted the complete remission of GIST until 110 days. The TGI rates of NN2101‐DM1 against imatinib‐resistant GIST and HMC1.2 cells were high. Furthermore, NN2101‐DM1 exhibited higher therapeutic efficacy against SCLC cells than carboplatin/etoposide. The combination of NN2101‐DM1 and carboplatin/etoposide exerted synergistic growth‐inhibitory effects against SCLC cells. These results suggest that NN2101‐DM1 is a potential therapeutic for c‐Kit‐positive cancers irrespective of their c‐Kit mutational status.

## Conflict of interest

The authors declare no conflict of interest.

## Author contributions

SGP has full access to the data and takes responsibility for the integrity of the data and the accuracy of the analysis. J‐OK contributed to methodology, validation, formal analysis, and investigation. K‐HK contributed to validation, formal analysis, and investigation. EJB contributed to formal analysis and investigation. BP contributed to formal analysis and investigation. MKS contributed to formal analysis and investigation. BJK contributed to methodology, validation, and formal analysis. H‐JK contributed to formal analysis and investigation. SGP contributed to methodology, formal analysis, supervision, project administration, funding acquisition, and writing—original draft.

### Peer review

The peer review history for this article is available at https://publons.com/publon/10.1002/1878‐0261.13084.

## Supporting information


**Fig. S1.** Expression levels of c‐Kit in various cell lines.
**Fig. S2.** NN2101 inhibits SCF‐mediated c‐Kit phosphorylation and its down‐stream signaling.
**Fig. S3.** Flow cytometry analysis of c‐Kit expression in various cells.
**Fig. S4.** Determination of specificity of NN2101 in cell binding.
**Fig. S5.** Internalization of NN2101 by various cancer cell lines.
**Fig. S6.** Stability analysis of c‐Kit protein.
**Fig. S7.** *In vitro* cytotoxicity of NN2101 and NN2101‐DM1.
**Fig. S8.** Serum stability of NN2101‐DM1.
**Fig. S9.** *In vivo* analysis of anti‐tumor activity.
**Fig. S10.** Individual analysis of anti‐tumor activity.
**Fig. S11.** Body weight change analysis.
**Fig. S12.** *In vitro* CDC analysis of NN2101.
**Fig. S13.** *In vitro* ADCC analysis of NN2101.
**Fig. S14.** Cytotoxicity analysis of human PBMC.

## Data Availability

The data reported in this study are available from the corresponding author (sgpark@ajou.ac.kr) upon reasonable request.
