# Supplementary material for: A novel anti‐c‐Kit antibody–drug conjugate to treat wild‐type and activating‐mutant c‐Kit‐positive tumors
Source: Mol Oncol. 2021 Aug 29;16(6):1290–308. doi: 10.1002/1878-0261.13084 (PMC8936518; doi:10.1002/1878-0261.13084)
Supplement: Supplementary file 1 — Fig. S1. Expression levels of c‐Kit in various cell lines. Fig. S2. NN2101 inhibits SCF‐mediated c‐Kit phosphorylation and its down‐stream signaling. Fig. S3. Flow cytometry analysis of c‐Kit expression in various cells. Fig. S4. Determination of specificity of NN2101 in cell binding. Fig. S5. Internalization of NN2101 by various cancer cell lines. Fig. S6. Stability analysis of c‐Kit protein. Fig. S7. In vitro cytotoxicity of NN2101 and NN2101‐DM1. Fig. S8. Serum stability of NN2101‐DM1. Fig. S9. In vivo analysis of anti‐tumor activity. Fig. S10. Individual analysis of anti‐tumor activity. Fig. S11. Body weight change analysis. Fig. S12. In vitro CDC analysis of NN2101. Fig. S13. In vitro ADCC analysis of NN2101. Fig. S14. Cytotoxicity analysis of human PBMC. [file MOL2-16-1290-s001.pdf]

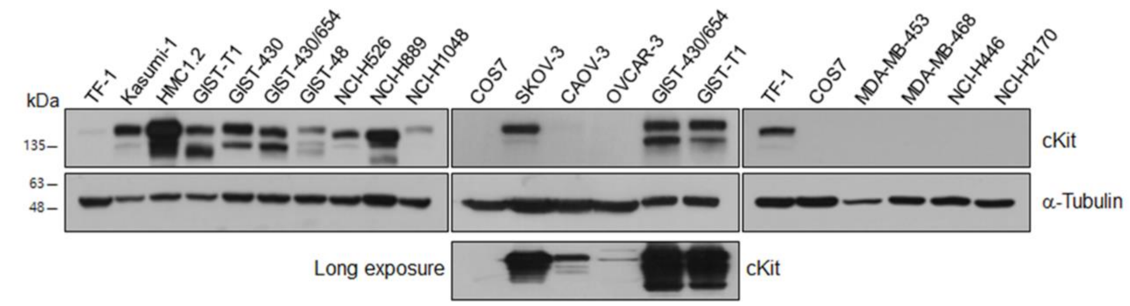

Figure S1. Expression levels of c-Kit in various cell lines. Expression levels of c-Kit protein were assessed in various cancer cell lines by performing western blot. COS7, MDA-MB-453, MDA-MB-468, NCI-H446, and NCI-H2170 were used as c-Kit-negative control cells. Tubulin was used as a loading control.

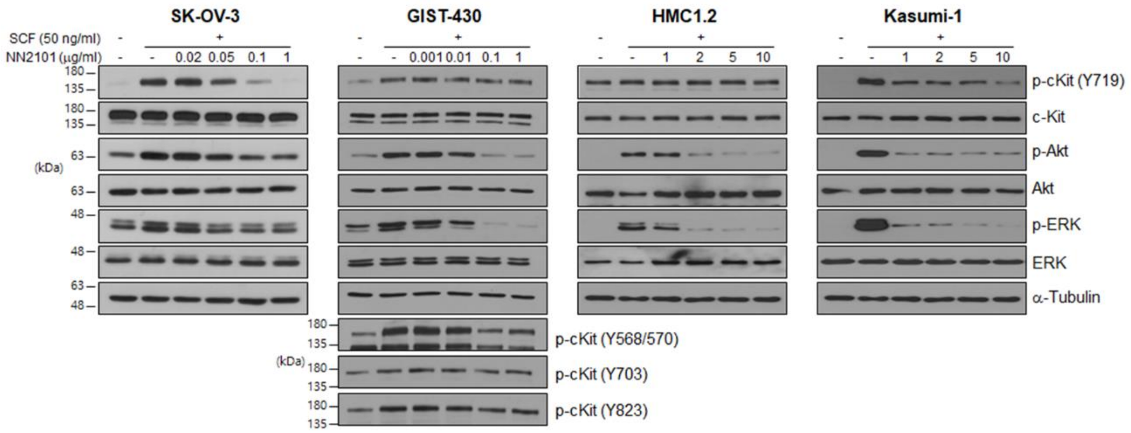

Figure S2. NN2101 inhibits SCF-mediated c-Kit phosphorylation and its down-stream signaling. Cells were serum starved for 6 h, then pretreated for 1 h with the indicated concentration of NN2101, with subsequent stimulation using SCF. All experiments were independently repeated at least three times. Tubulin was used as a loading control.

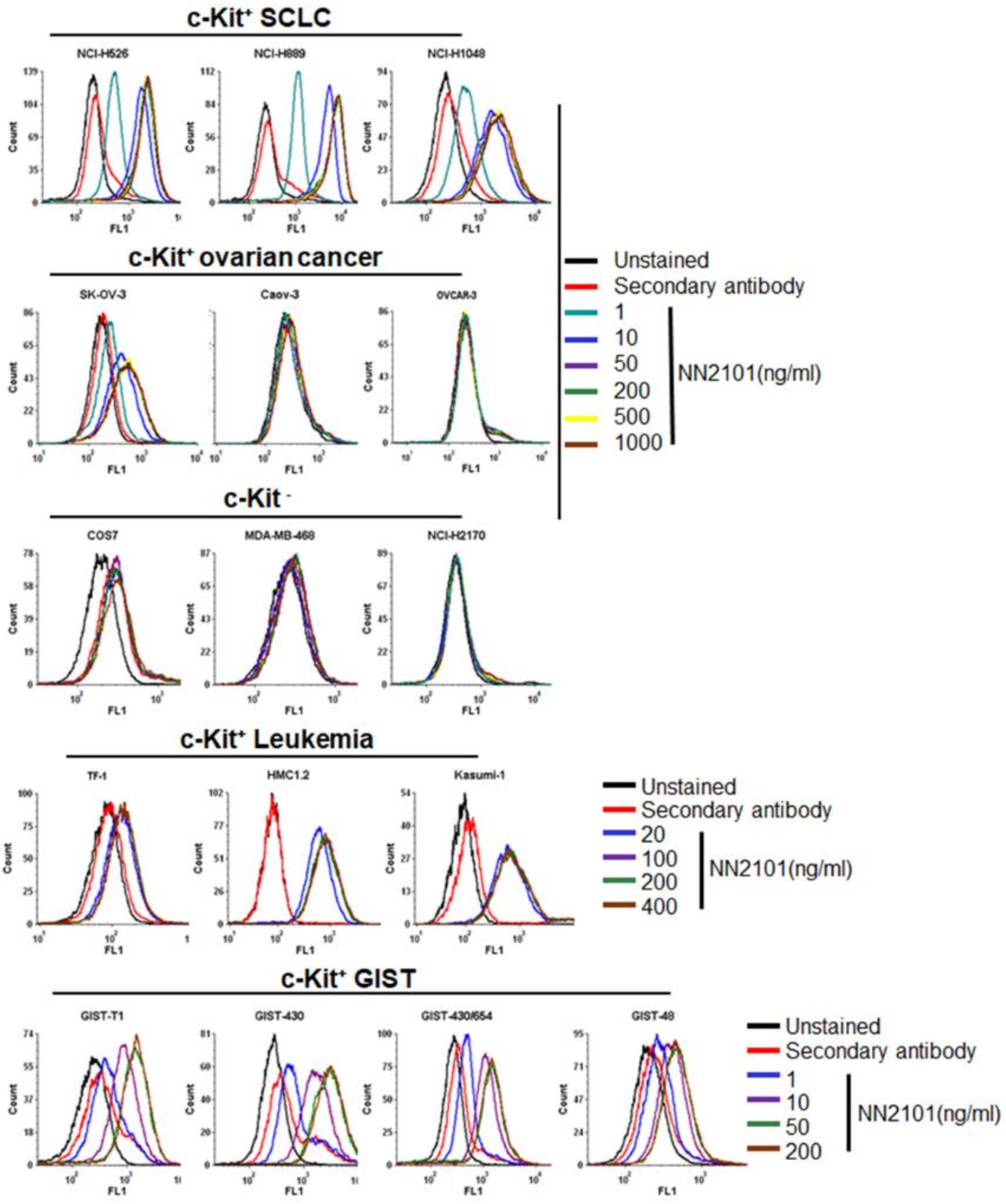

Figure S3. Flow cytometry analysis of c-Kit expression in various cells. Cells were treated with the indicated concentration of NN2101 and cell binding was analyzed by flow cytometry. COS7, MDA-MB-468, and NCI-H2170 were used as c-Kit negative cell lines.

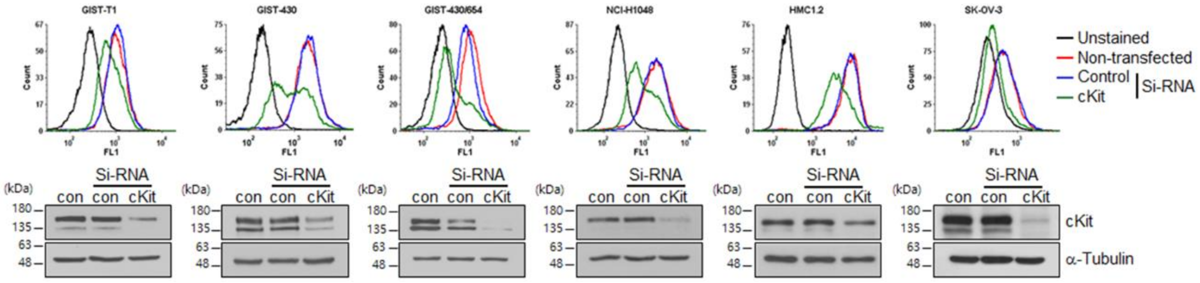

Figure S4. Determination of specificity of NN2101 in cell binding. Cells were transfected with 40 nM of control or c-Kit siRNA for 72 h. Knockdown of c-Kit was determined by western blot. Tubulin was used as a loading control.

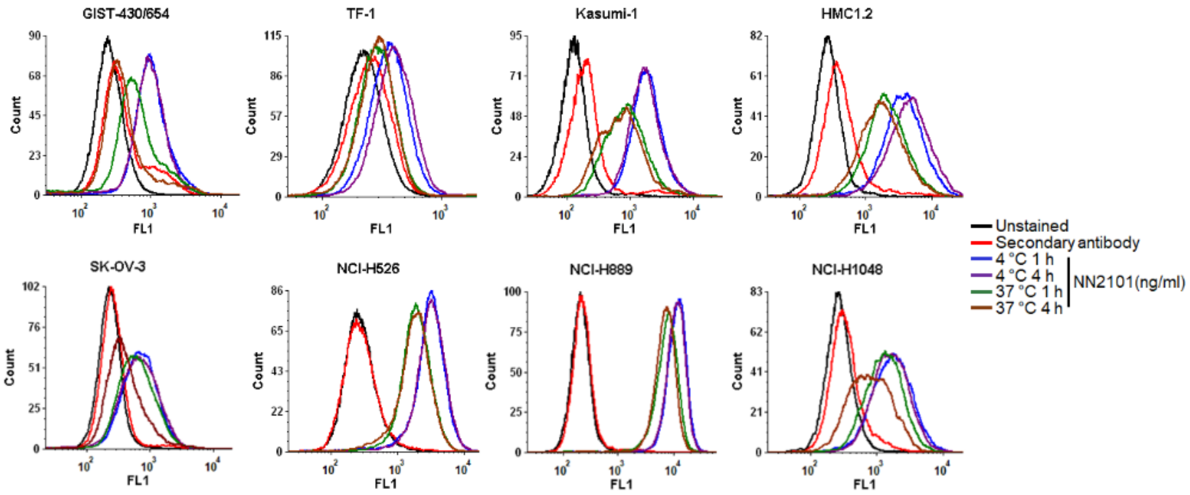

Figure S5. Internalization of NN2101 by various cancer cell lines. Cancer cells were incubated in the presence of cycloheximide (75 μg/ml) and blocked with Fc blocker for 10 min to preclude Fc receptor-mediated internalization. Cells were then incubated in the presence or absence of NN2101 (1 μg/ml) at 4°C or 37°C for 1 - 4 h and then analyzed by flow cytometry.

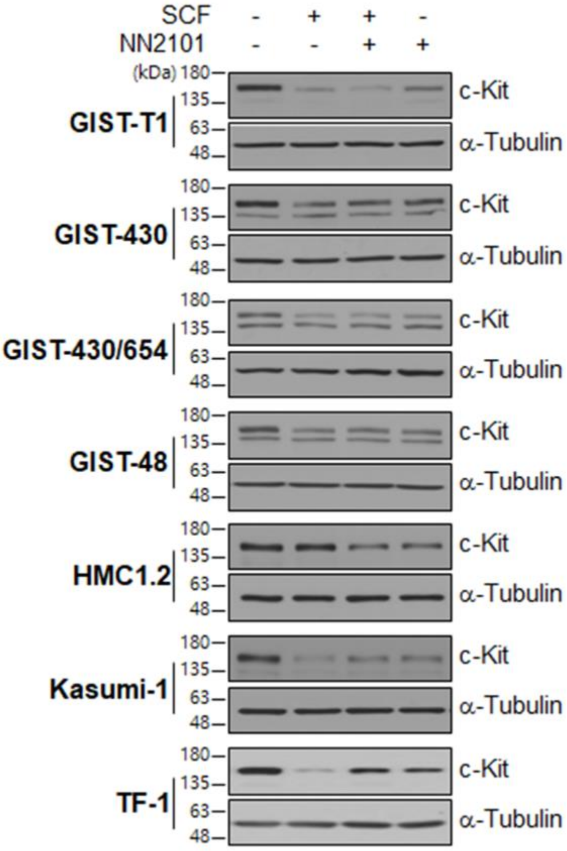

Figure S6. Stability analysis of c-Kit protein. Cells were cultured in media containing 10% FBS and treated with SCF (100 ng/ml), NN2101 (5 μg/ml), or a combination of SCF with NN2101 for 24 h. The expression levels of c-Kit protein were determined by western blot. The experiments were independently repeated at least three times. Tubulin was used as loading control.

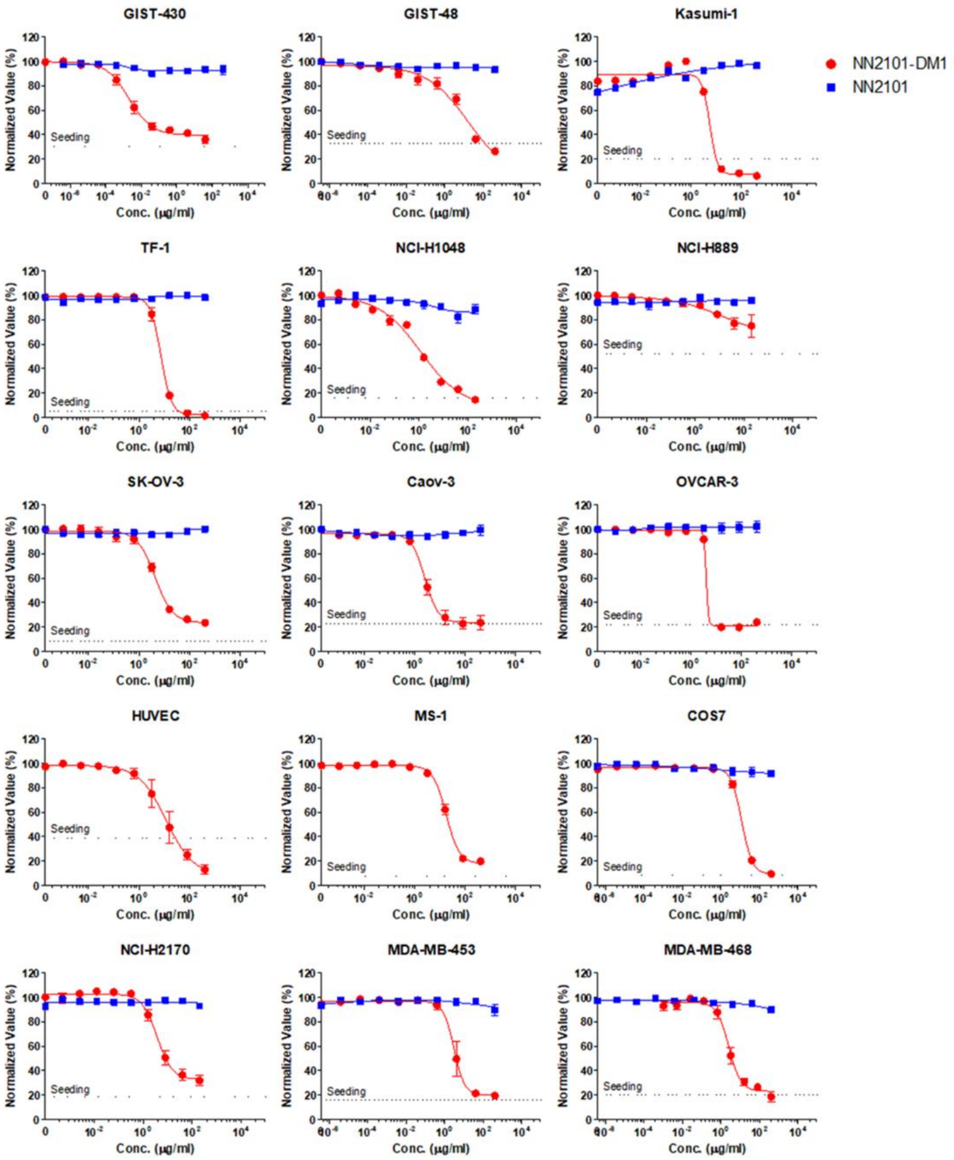

Figure S7. *In vitro* cytotoxicity of NN2101 and NN2101-DM1. Cells were cultured in their respective media and treated with NN2101 or NN2101-DM1 for 4-5 days in a dose-dependent manner. Cytotoxicity was analyzed as described in the Methods section. All experiments were independently repeated at least three times.

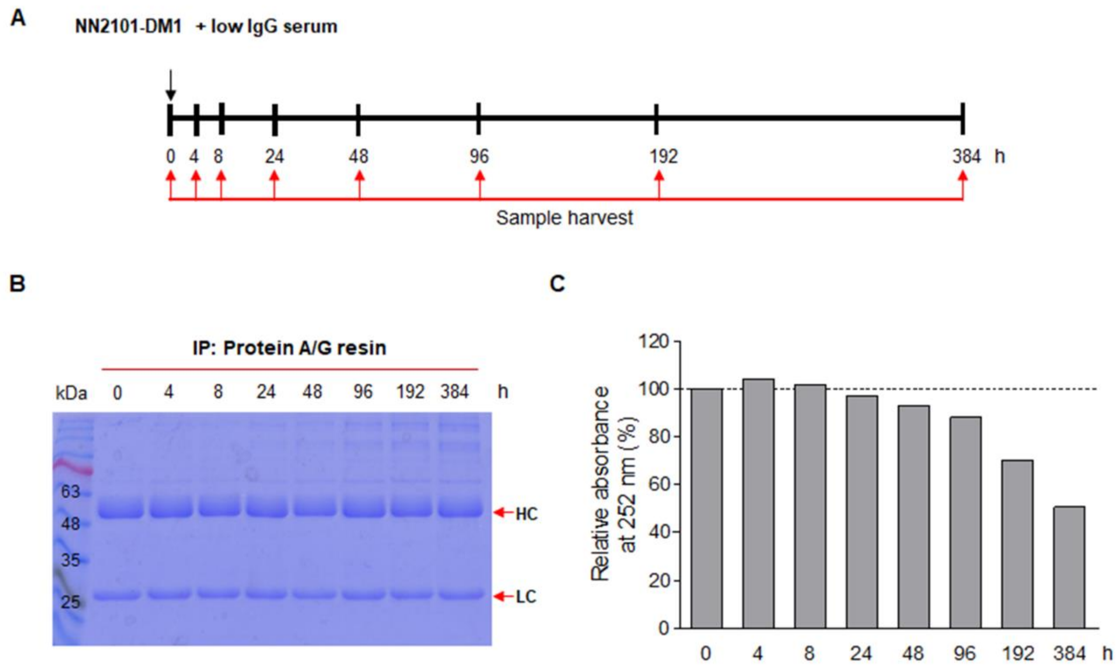

Figure S8. Serum stability of NN2101-DM1. NN2101-DM1 was incubated in low IgG serum as indicated (A) and precipitated with protein A/G agarose as described in the Methods section (B). Then, optical absorbance at 252 nm was normalized and presented (C). HC and LC indicate heavy chain and light chain, respectively.

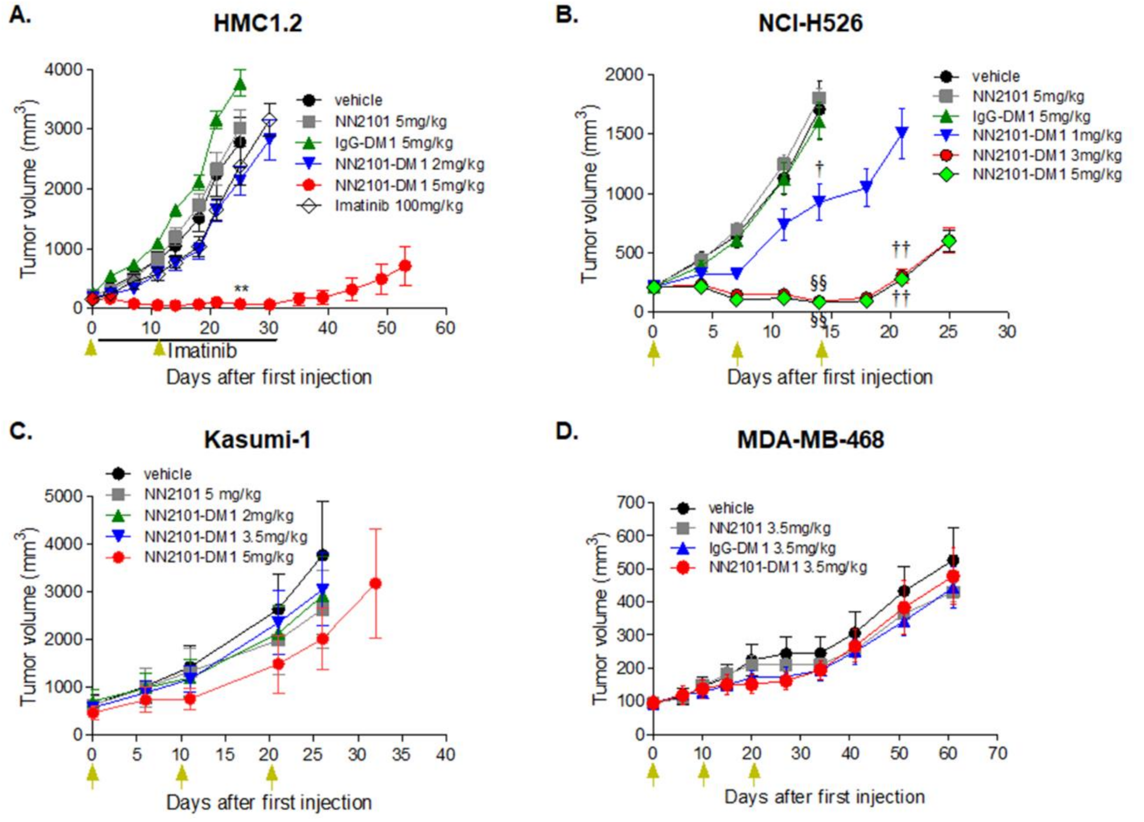

Figure S9. *In vivo* analysis of anti-tumor activity. Cells were implanted into immune deficient mice (n= 6 - 7) as described in the Methods section. Vehicle, NN2101, IgG-DM1, or NN2101-DM1 was intravenously administered (arrow) and imatinib was orally administered once a day for 30 days. † vs. vehicle, NN2101, and IgG-DM1; ** vs. vehicle, NN2101, imatinib, IgG-DM1, and NN2101-DM1 2 mg/kg; §§ vs. vehicle, NN2101, IgG-DM1, and NN2101-DM1 1 mg/kg; †† vs. NN2101-DM1 1 mg/kg. † $P < 0.05$, ** $P < 0.01$, §§ $P < 0.01$, and †† $P < 0.01$.

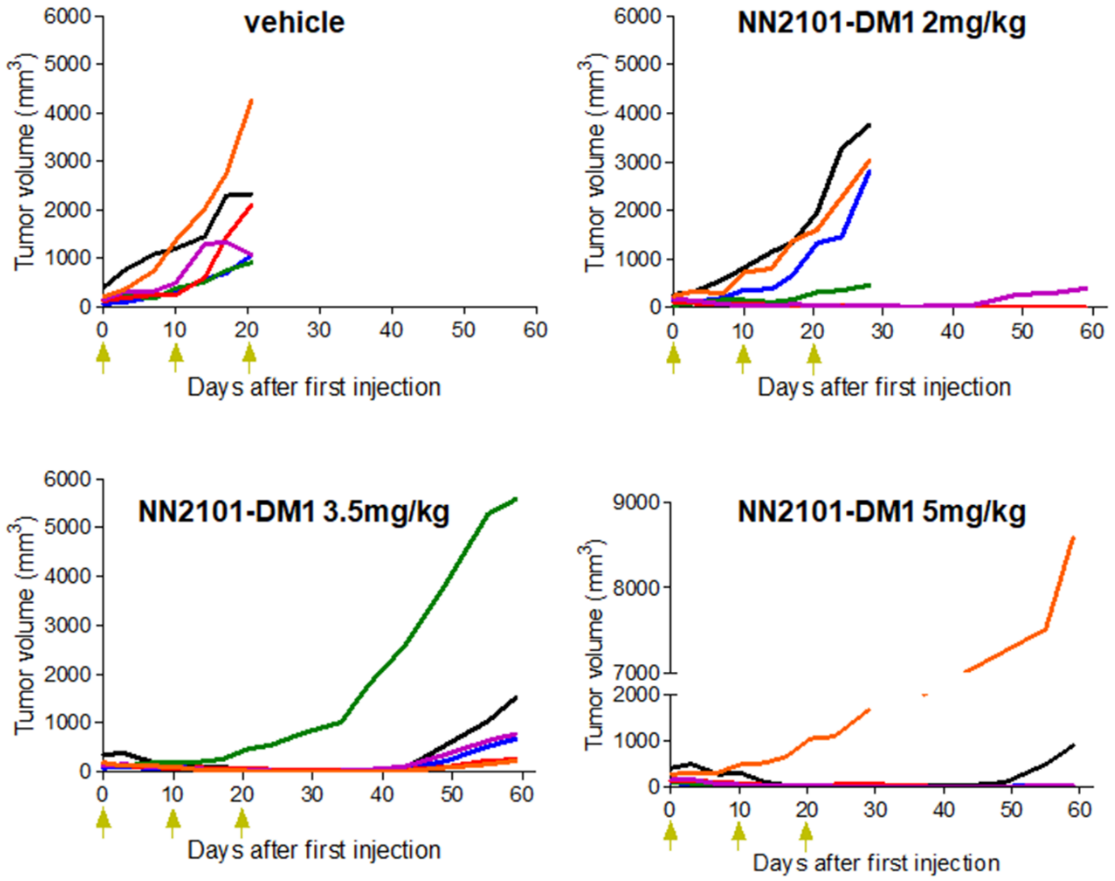

Figure S10. Individual analysis of anti-tumor activity. The results shown in figure 6D are presented for each individual (n = 6).

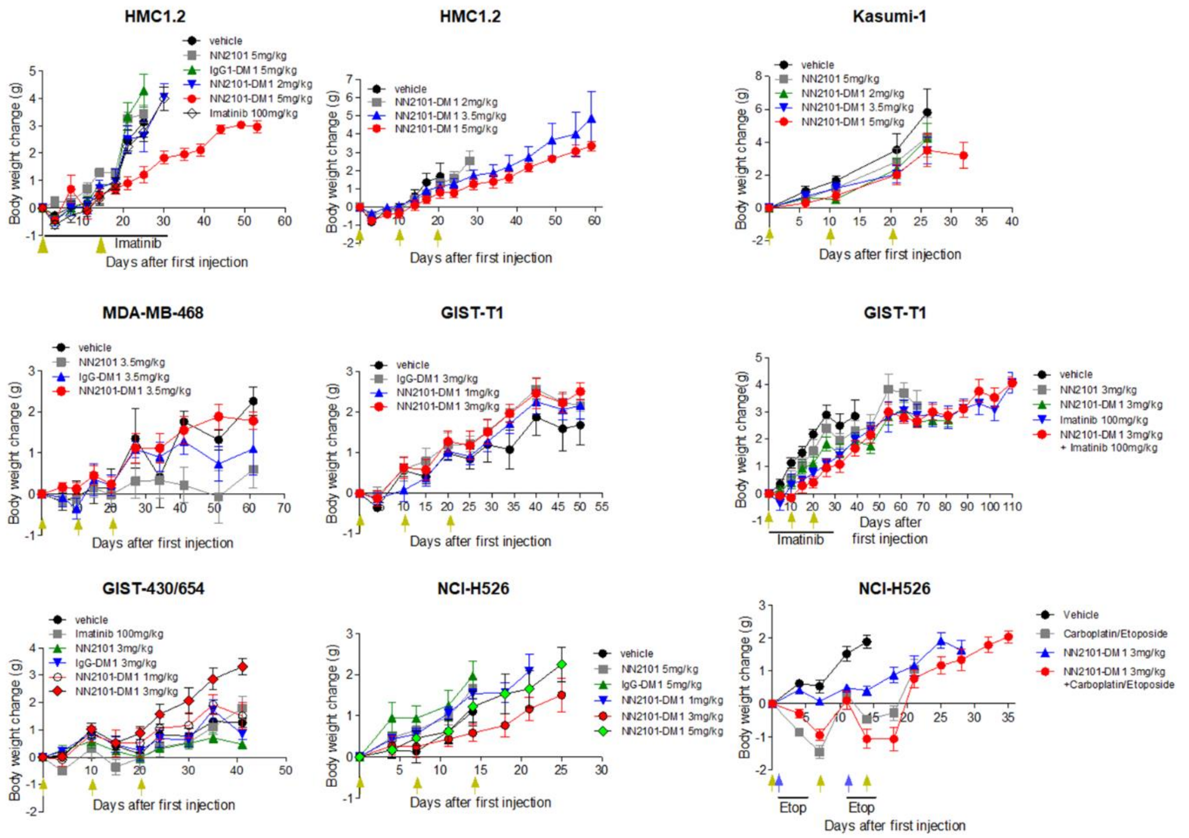

Figure S11. Body weight change analysis. Body weight changes were monitored every 3-5 days after drug administration. The green arrow indicates administration of vehicle, NN2101, IgG-DM1, or NN2101-DM1, and the blue arrow indicates administration of carboplatin.

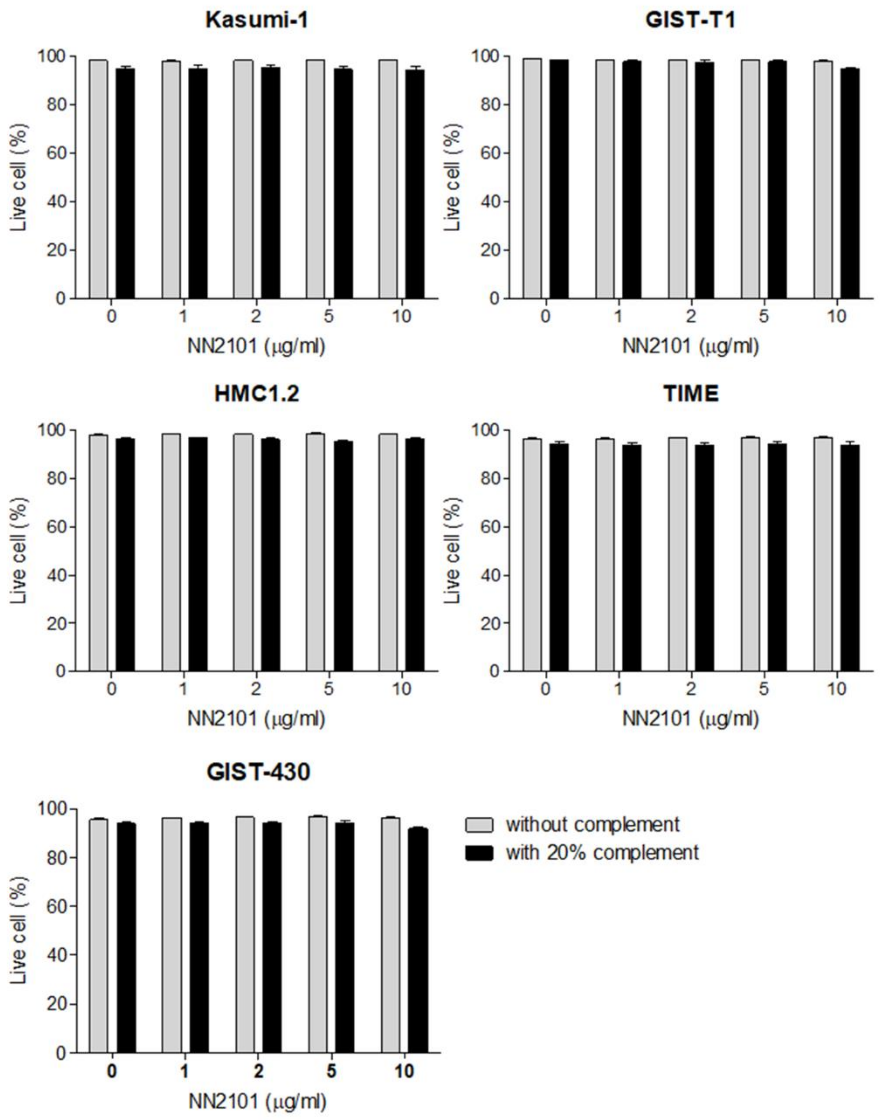

Figure S12. ***In vitro*** **CDC analysis of NN2101.** CDC activity was examined using various cells as indicated in the presence or absence of 20% human serum (v/v) for 4 h. Viable cells were directly counted using Calcein AM/Hoechst 33342 staining as described in the Methods section. The results represent means ± SEM of three independent experiments.

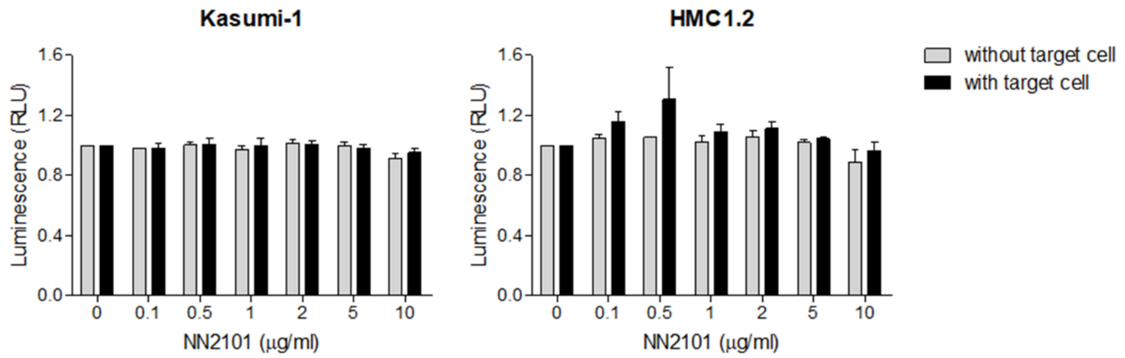

Figure S13. ***In vitro*** **ADCC analysis of NN2101.** ADCC activity was examined using Kasumi-1 and HMC1.2 cells (target cells) in the presence of *Jurkat* cells stably expressing the FcγRIIIa receptor (V158 variant), and an NFAT-response element driving expression of firefly as effector cells (effector/target ratio = 6:1) with the indicated concentration of NN2101 for 6 h.

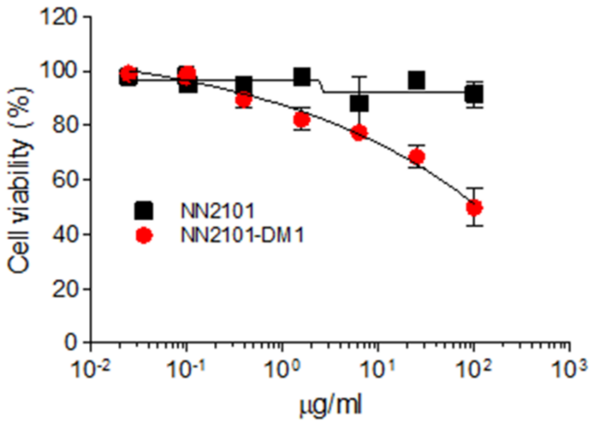

Figure S14. Cytotoxicity analysis of human PBMC. NN2101 or NN2101-DM1 were used to treat human PBMC (n = 3) in a dose-dependent manner for 4 days. Live cells were stained with Hoecst 33342 (10 μM) and Calcein AM (1.2 μg/ml) at 37°C for 30 min and quantitated using a Celigo Imaging Cytometer. The results represent means ± SEM of at least three independent experiments.
